# Activating Carbon and Oxygen Bonds for Low-Temperature Thermal Decomposition of Spent Lithium-Ion Battery Cathode Materials

**DOI:** 10.1021/acs.est.4c12200

**Published:** 2025-03-06

**Authors:** Kang Liu, Xiaohong Zhu, Yuying Zhang, Mengmeng Wang, Roya Maboudian, Daniel S. Alessi, Daniel C.W. Tsang

**Affiliations:** a Department of Civil and Environmental Engineering, The Hong Kong University of Science and Technology, Clear Water Bay, Hong Kong 999077, China; b Department of Civil and Environmental Engineering, University of California Berkeley, Berkeley, California 94720, United States; c Department of Chemical and Biomolecular Engineering, University of California Berkeley, Berkeley, California 94720, United States; d Department of Earth and Atmospheric Sciences, University of Alberta, Edmonton, Alberta T6G 2E3, Canada

**Keywords:** retired NCM cathode, waste pyrolysis, critical metal recycling, carbon−mineral interactions, resource circularity, carbon emission reduction

## Abstract

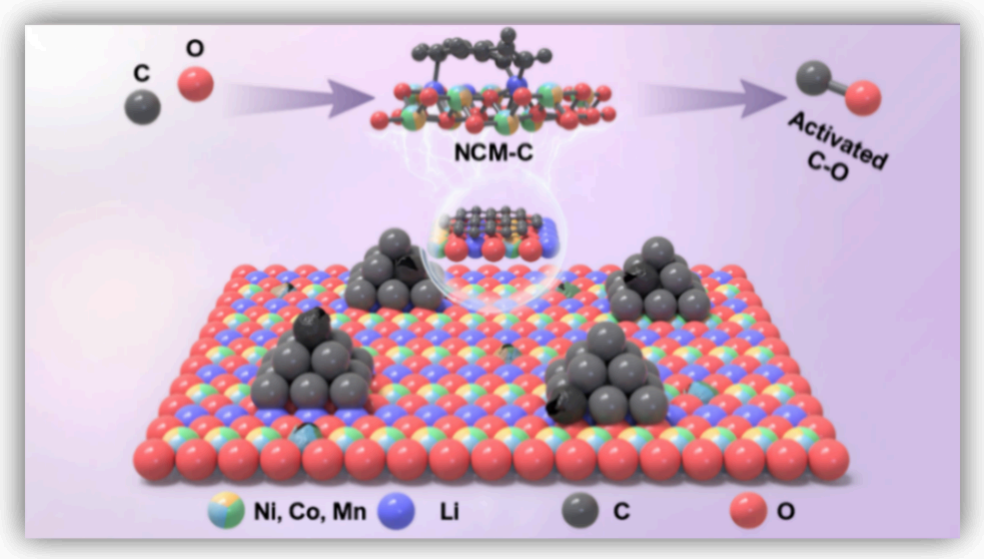

The temperature for complete disintegration of spent lithium-ion battery (LIB) cathode materials is typically in a range of 750–1400 °C, resulting in intensive energy consumption and high carbon emissions. Here, we promote the bond activation of oxygen in LiNi_0.5_Co_0.2_Mn_0.3_O_2_ and carbon in graphite electrodes, achieving rapid gasification and thermal decomposition of active crystals at lower temperatures in the absence of other activating agents. The activation of C and O bond leads to the storage of internal energy and the transition of the crystalline phase (single crystal to polycrystal) of the active crystals. Density functional theory modeling confirms that the CO adsorption energy is significantly higher with C_*a*_–O_*a*_ (−3.35 eV, C and O activation) than with no activation (−1.66 eV). The differential charge results show that the bond activation model has the highest charge accumulation and consumption, improving the electron transfer. The Bader charge transfer between C_*a*_–O_*a*_ and CO is also the largest, with a value of 0.433 |e|. Therefore, synchronous activation of C and O bonds can reduce the decomposition temperature of active crystals by 200 °C and allows a low-temperature pyrolysis recycling of retired LIB cathode materials. Our research provides a potential strategy for low-carbon recycling of retired LIBs worldwide.

## Abstract 2

Low-temperature pyrolysis of spent lithium-ion battery cathode materials can be achieved by synchronously activating the C and O bonds to promote gas adsorption and charge transfer.

## 1 Introduction

Electric vehicles are considered the primary choice for transport decarbonization.^1,2^ When compared to a gasoline-powered vehicle, a single electric vehicle emits only 22.4 tonnes of carbon over its entire lifecycle, representing a 43.4% reduction.^3^ Power lithium-ion batteries (LIBs) are currently the key components of electric vehicles, accounting for 40–60% of overall vehicle costs.^4,5^ Active cathode materials are critical components influencing the performance and market prices of power LIBs.^6,7^ More than half of the electric vehicle market share is represented by active cathode materials composed of transition metal oxide layered structures (e.g., LiCoO_2_, LiNi*_*x*_*Co*_*y*_*Mn_1–*x–y*_O_2_, and LiMn_2_O_4_) based on lithium, due to their high energy density and long endurance compared to lithium iron phosphate (LiFePO_4_).^8,9^ Consequently, the prevalent utilization of power LIBs is highly dependent on the sustainable supply of critical metals (e.g., Li, Ni, Co, and Mn).^10,11^

Pyrometallurgical processing,^12,13^ hydrometallurgical separation,^14,15^ and material regeneration^16,17^ are recognized as the three primary pathways for recycling the retired LIBs. Owing to its high reactivity, lithium is susceptible to depletion in the complex and time-consuming hydrometallurgical processes, resulting in a low recovery rate of ∼50% and a limited profit.^18,19^ In pyrometallurgical processing, the high-temperature escape of framework oxygen from the active cathode materials (e.g., LiCoO_2_, LiNi*_*x*_*Co*_*y*_*Mn_1–*x–y*_O_2_, and LiMn_2_O_4_) results in the selective release of lithium and destabilization of crystal structures (e.g., layered spinel and rock salt phases),^20^ allowing for the preferential extraction of lithium in engineering practices.^21,22^ The active cathode materials of lithium-transition metal oxide-based structures possess high thermochemical stability, which is imperative for the durable operation of LIBs.^23,24^ However, this feature poses challenges in pyrometallurgical recovery.^25,26^ Pyrometallurgical processing of retired lithium-ion batteries (LIBs) typically requires temperatures above 750–1400 °C.^27,28^ These thermodynamic characteristics require a high energy input for the recovery systems of retired LIBs.^29,30^ The high costs of metal extraction and possibility of carbon escape harm the sustainable prospects of transport decarbonization by an electric vehicle.^30,31^ Developing low-temperature, low-carbon, and cost-effective strategies for deconstructing active cathode materials can promote closed-loop recycling of the retired LIBs.^32,33^

The interfacial reduction of transition metals triggered by a variety of reducing agents is the focus of current research on the disintegration of active cathode materials.^34,35^ The gasification reduction of transition metal–oxygen octahedra within the active crystal framework facilitates a decrease in the valence states of transition metals (e.g., Ni^2+^, Co^3+^, and Mn^4+^), accelerating lithium release and phase transformation.^36,37^ Carbon-based reductants can be a preferred option for in situ interfacial reduction of active cathode materials in pyrometallurgical processing.^38,39^ The temperature range for the reduction of LiCoO_2_ active crystals in the presence of graphite (1000 °C) is significantly lower than their self-decomposition temperature (1400 °C).^40,41^ However, high pyrolysis temperatures unavoidably result in significant energy consumption and carbon emissions.^42^

In this study, we developed a practical technique for accomplishing the low-temperature pyrolysis of retired LIB cathode materials. First, the LiNi_0.5_Co_0.2_Mn_0.3_O_2_ (NCM) phase was activated with graphite electrode material (5 wt % C addition) during which the C and O bonds of NCM-C could be activated to increase the reaction activity and internal energy storage of mixed materials. The O in NCM and C in graphite led to rapid production of CO that can influence the Boudouard reaction equilibrium and hasten the low-temperature transition of NCM from the spinel phase to transition metal oxides. According to density functional theory (DFT) results, an electronic configuration established by C and O bond activation can enhance the charge transfer and CO adsorption for thermal decomposition. The life cycle assessment (LCA) results show that the activation of C and O bonds of NCM-C materials can significantly reduce pollutant emissions and carbon escape, thus mitigating the global environmental impact during the recycling process of retired LIBs.

## 2 Materials and Methods

### 2.1 Materials and Reagents

A variety of chemical reagent providers were used to purchase the following: nitric acid (HNO_3_, AR, 65–68%, Aladdin), sulfuric acid (H_2_SO_4_, AR, 98%, Aladdin), and hydrochloric acid (HCl, AR, 36–38%, Aladdin). The spent LIB ternary powder (LiNi_0.5_Co_0.2_Mn_0.3_O_2_) was purchased from Xiaopeng New Energy Vehicle Company in Guangdong, China., via the online marketplace for used goods (Alibaba). Tables S1 and S2 provide the pertinent spent powder LIB material parameters. The compositions of Li, Ni, Co, and Mn in the cathode material powder (LiNi_0.5_Co_0.2_Mn_0.3_O_2_) were determined using the mineral acid digestion procedure, and deionized (DI) water was utilized.^43^

### 2.2 Experimental Procedure

A three-step recycling procedure was employed in the intended spent LIB recycling experiment, which involved disassembling the battery, activating the C and O bonds, and pyrolyzing the ball-milled cathode materials at high temperatures.

1) Battery disassembly: To liberate surplus energy and avoid potential self-ignition and explosive reactions, the exhausted LIBs were disassembled using an improvised makeshift lighting mechanism. In a vented fume hood, the spent powder LIBs were then manually separated, producing cathode electrode plates, anode electrode plates, separators, plastic casings, and electrolytes. The copper foil and graphite from the anode electrode plates were directly separated and recovered using an ultrasonic device with only clean DI water as the medium. The cathode electrode plates were broken into smaller pieces using a universal grinder (made by Madsen Pharmaceutical Machinery Factory in Shandong Province, China), and the cathode electrode material powder (LiNi_0.5_Co_0.2_Mn_0.3_O_2_) was separated from the coiled aluminum foil using a 200-mesh sieve.
2) C and O bond activation: In a zirconia ball milling jar, powdered cathode electrode material and detached graphite from anode electrode plates were mixed in varying mass ratios (0, 5, 10, 15, and 20 wt %). A high-energy planetary ball mill (DECO-PBM-AD-0.4L, Deco Technology Development Co. Ltd., Changsha, China) was then used to place the sealed jar to perform a reaction at various rotational speeds (0, 200, 400, 600, and 800 rpm), with the reaction time set at 12 h under room temperature and normal pressure. The powder sample, activated through mechanochemical processing, was taken after the reaction and used as an experimental sample for pyrolysis.
3) Pyrolysis reaction: A pyrolysis reaction was carried out on the C and O bond activation sample by placing it in a quartz boat and introducing it to a tube furnace. In this reaction, N_2_ served as the protective gas, and the ultimate temperatures were set at 450, 550, and 650 °C with a regulated heating rate of 5 °C/min. The pyrolysis holding time was universally set to 1 h. Following the completion of the pyrolysis reaction, a sample of the powder was dissolved in deionized water, and a 0.45 mm membrane was used to extract the filtrate. The release efficiency of Li from the LiNi_0.5_Co_0.2_Mn_0.3_O_2_ phase was then calculated by measuring the Li content in the deionized water using an inductively coupled plasma optical emission spectroscopy (ICP-OES, SPECTRO ARCOS, Germany).

### 2.3 Analytical Methods

The phase transition of LiNi_0.5_Co_0.2_Mn_0.3_O_2_ at high temperatures was closely related to the Li release efficiency. According to the following formula, the release efficiency of Li in the LiNi_0.5_Co_0.2_Mn_0.3_O_2_ phase can be determined:
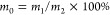
IHere, *m*_0_ denotes the fraction of Li that was released, *m*_1_ denotes the amount of Li that was dissolved in deionized water, and *m*_2_ is the amount of Li that was originally present in the LiNi_0.5_Co_0.2_Mn_0.3_O_2_ phase, as measured by the mineral acid digestion method (Note S1).

### 2.4 Material Characterization

A laser particle size analyzer (Malvern Master sizer 3000 Laser Particle Size Analyzer, UK) was used to demonstrate the particle size distribution of mixed NCM-C materials. Scanning electron microscopy (SEM, JEOL model JSM-6490, Japan) was used to analyze the micromorphology of the mixed materials, providing information about elemental distribution mapping (EDS). Titan G2 60-300 (HR-TEM-Mapping) apparatus with an image corrector, produced by FEI in the United States, was used to perform high-resolution transmission electron microscopy-mapping measurements. Using a TESCAN VEGA3 XM instrument from the Czech Republic, the surface morphology of the items was investigated by using scanning electron microscopy with energy-dispersive X-ray analysis (SEM-EDAX). Aberration-corrected, high-angle annular dark field scanning transmission electron microscopy (AC HAADF-STEM, FEI G2 60-300 with ChemiSTEM Technology, U.S.A.) was used to characterize the microscale changes in the mixed NCM-C materials including crystal structure, grain boundary penetration, crystal defect, lattice distortion, and element distribution. Supplementary characterization methods are provided in Note S2. The life cycle assessment (LCA) calculation method is provided in Note S3.

### 2.5 Theoretical Calculations

The Vienna Ab Initio Simulation Package program, based on a plane wave basis set and pseudopotentials, was used to carry out the density functional theory calculations.^44^ With the generalized gradient approximation, the exchange–correlation effects between the electrons and atoms were considered. The generalized gradient approximation Perdew–Burke–Ernzerhof function was used.^45^ The Brillouin zone integration was carried out using the Monkhorst-Pack special *k*-point sampling method, with the electronic wave functions enlarged in plane waves.^46^ For the structural relaxation and static calculations, a 3 × 3 × 1 *k*-mesh centered at the Γ point was used. A vacuum layer with a size of 12 Å was introduced in the *z*-direction to ensure minimal interaction between the layers of the materials in different periodicities. All calculations considered spin polarization of electrons and utilized periodic boundary conditions. The convergence threshold for both ionic and electronic self-consistent iterations was set to 1 × 10^–5^ eV.

## 3 Results and Discussion

### 3.1 Structural Characteristics of NCM-C Materials

**Figure 1:**
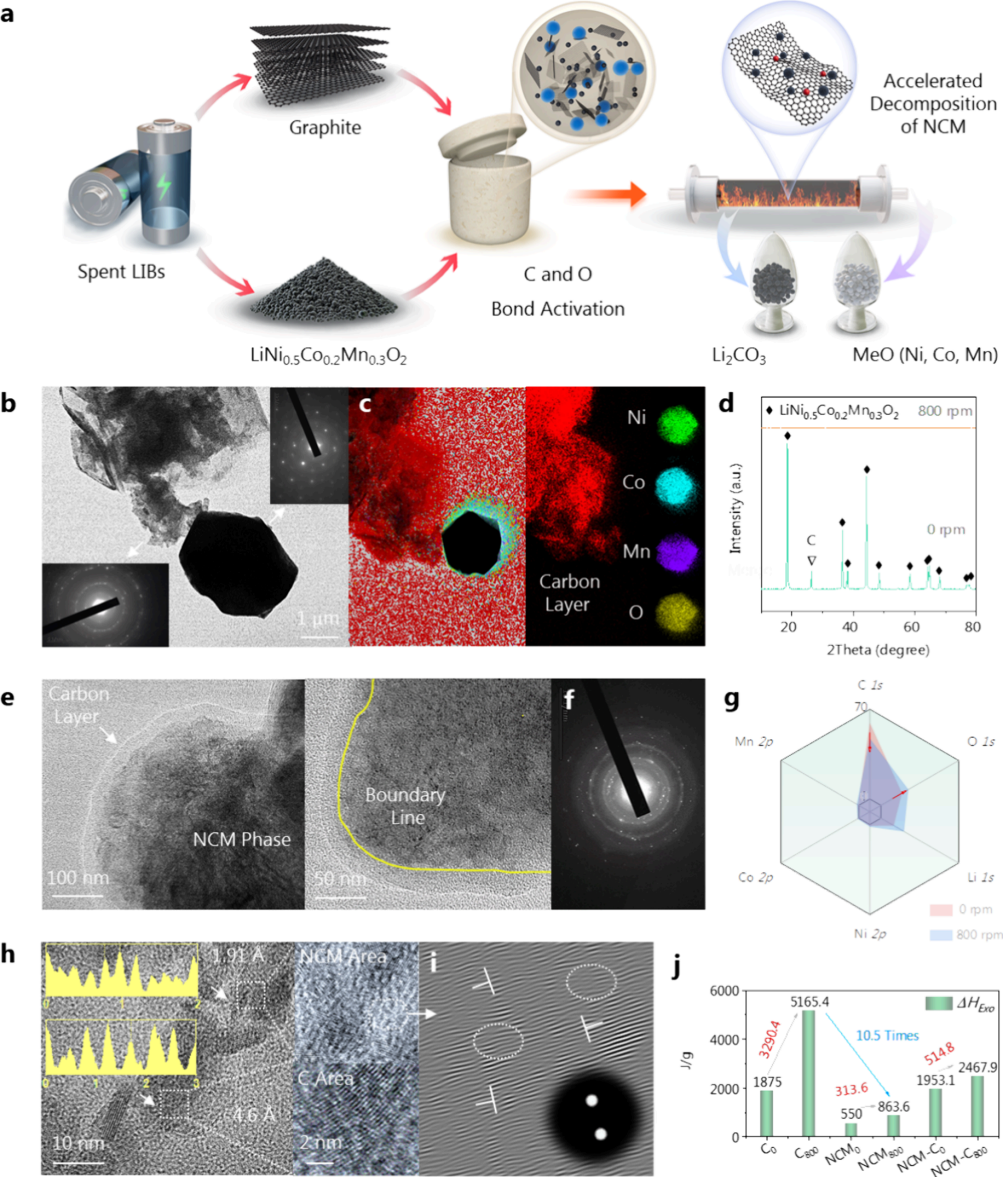
(a) Schematic diagram of the synchronous activation of C and O bonds in the spent LIBs; characterization of NCM-C mixed materials before (0 rpm) and after (800 rpm) C and O bond activation: (b) HRTEM-SAED of 0 rpm sample; (c) merged (left) and individual elements (right) EDS mapping on the 0 rpm sample; (d) XRD patterns before and after C and O bond activation; (e) HRTEM results of NCM-C mixed materials after C and O bond activation (at 100 and 50 nm scales); (f) SAED results corresponding to panel (e); (g) XPS-based near surface elemental contents of NCM-C mixed materials before and after C and O bond activation; (h) lattice measurement of NCM-C mixed (800 rpm) material, where the NCM area represents the O region and C area represents the graphite region; (i) local lattice amplification of panel (h); (j) internal energy measurement of C, NCM, and NCM-C mixed materials before (the subscript is 0 rpm) and after (the subscript is 800 rpm) C and O bond activation, where the internal energy results are integrated by fitting the DSC curve with the time parameter (in s).

The scheme of the activation of the C and O bonds in the spent LIBs is shown in Figure 1a. When the NCM-C mixture was subjected to high-energy mechanochemical forces, the C bonds of graphite and the O bonds of NCM were activated to form C–O bonds and achieve energy conservation. Using the high-resolution transmission electron microscope (HRTEM) examination and the selected area electron diffraction (SAED) findings, we verified that NCM is a single crystal, while graphite is polycrystalline, and the white arrows in Figure 1b indicate this distinction. The elliptical NCM particles were distributed in various areas (Figure S1), and there was a significant decrease in the particle size (Figure S2). It is worth noting that graphite does not invade the crystal structure of NCM (0 rpm sample, Figure 1c). Figure 1d demonstrates that the diffraction peaks of graphite and LiNi_0.5_Co_0.2_Mn_0.3_O_2_ at 28.8° vanish entirely, highlighting that the C and O bond activation on NCM-C mixed materials results in distortion of the crystal lattice. Under higher magnification, a carbon coating layer deposits onto the NCM, and a clear demarcation line between graphite and NCM crystal phases is observed (Figure 1e). Figure 1f confirms that the treated NCM-C mixed materials are polycrystalline, as evidenced by the polycrystalline diffraction rings. The corresponding scanning electron microscopy (SEM) and energy-dispersive spectroscopy (EDS) results in Figure S3 reveal the presence of spherical NCM particles in the 0 rpm sample. Following the C and O bond activation, the 800 rpm sample exhibits the conversion of NCM particles into sheet-like formation. Simultaneously, the elemental distribution of Ni, Co, Mn, and O elements is obscured, leaving only a high concentration distribution of the C element visible, suggesting that the graphite material has effectively encapsulated NCM oxides.

X-ray photoelectron spectroscopy (XPS) was used to investigate the near-surface components of the NCM-C mixed materials (Figure 1g) and individual elements for comparison (Figures S4 and S5). Following the C and O bond activation, there was a notable rise in the surface content of carbon species, accompanied by a large decrease in the surface content of O (Figure S4). This suggests the encapsulation of C on the NCM crystal phase, consistent with Figure 1e. Ni, Co, and Mn species are reduced to varying degrees in the solid state due to the interactions with graphite (Figure S5). In the HRTEM image, when observing at a scale of 10 nm, an amorphous–crystalline hybrid boundary region is observed between NCM and C (Figure 1h). The enlarged image reveals the distortion of lattice stripes in the NCM region (Figure 1i), providing evidence of the destruction of the oxides. Internal energy is stored in the crystals in the form of lattice distortion.

Additional computations can determine the internal energy storage capacity of NCM and graphite (Figure 1j). Following the C and O bond activation, the energy storage capacity (Figure S6) of graphite increased significantly from 1875 J/g (0 rpm) to 5165.4 J/g (800 rpm), whereas that of NCM only increased from 550 J/g (0 rpm) to 863.6 J/g (800 rpm). The internal energy storage of graphite material was 10.5 times more than that of NCM crystals. The incremental internal energy of the NCM-C mixed materials increased from 1953.1 to 2467.9 J/g (800 rpm). When considering a quantitative mixing ratio of 5 wt % graphite and 95 wt % NCM, the mixed energy increment per unit interval was 514.8 J/g. This value was higher than the sum of the individual energy increments, which was 462.4 J/g, representing a difference of 52.4 J/g in enthalpy change, Δ*H* (Figure 1j). This quantitative outcome validates the collaborative characteristics of energy storage in NCM and graphite. The internal energy is stored in various forms within the active sites of the NCM-C mixed materials that can be leveraged in subsequent pyrolysis reactions. Furthermore, NCM and graphite crystals (Figure S7) exhibited grain refinement and amorphous morphology, while after thermochemical reactions, due to the release of internal energy from the lattice, NCM and C partially recovered their crystal structures.^47^

### 3.2 Activation of C and O Bonds

**Figure 2:**
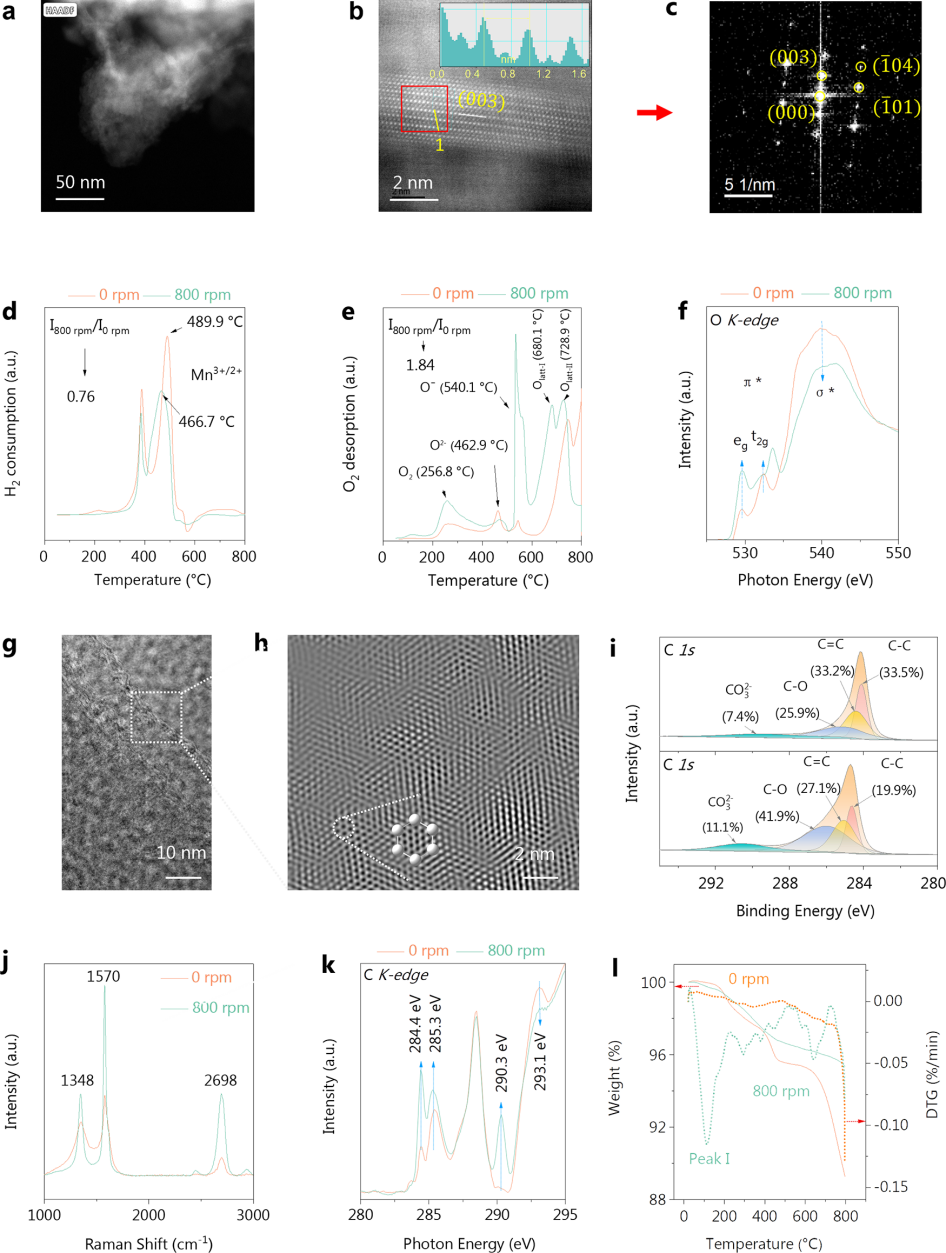
Characterization of O bond activation: (a) HAADF-STEM (50 nm), (b) STEM result (2 nm, the built-in image shows the lattice measurement results of Line 1), (c) diffraction spot after Fourier transform in panel (b) (red border); characterization results of NCM-C mixed materials before C and O bond activation: (d) H_2_-TPR, (e) O_2_-TPD, and (f) XANES of O species; characterization of C bond activation: (g) AC HAADF-STEM of 800 rpm sample, (h) enlarged area of Figure 2g (2 nm, selecting the corresponding diffraction spots (i.e., six spots) for inverse fast Fourier transform to obtain panel (h)), (i) XPS high-resolution spectra of C 1s, (j) Raman spectra of graphite electrode, (k) C K-edge spectra of NCM-C mixed materials, and (l) TG-DTG results of graphite electrode.

White crystalline areas, attributed to the NCM crystal materials, could be identified in Figure 2a using dark field imaging by aberration-corrected high-angle annular dark-field scanning transmission electron microscopy (AC HAADF-STEM). We conducted a magnification and measurement of the lattice fringes in the crystalline materials (Figure 2b) and discovered varying levels of atomic defects in Line 1 of the NCM-C mixed materials. The result (0.495 nm) was close to the standard spacing of the (003) crystal planes (0.47563 nm). Therefore, the observed area belongs to LiNi_0.5_Co_0.2_Mn_0.3_O_2_. The uneven faults in Line 1 are probably generated by point defects in NCM crystals, which can arise when O escaped from the metal atoms. Figure 2c displays the Fourier transform result of Figure 2b (red border), confirming the presence of LiNi_0.5_Co_0.2_Mn_0.3_O_2_. The electron paramagnetic resonance (EPR) and high-resolution XPS spectra of the O 1s (Figure S8) demonstrated the activation of the metal–oxygen bonds. This resulted in the emergence of a distinct peak at *g* = 2.004, which is associated with oxygen vacancies (Figure S8a). XPS analysis (Figure S8b) shows that the surface concentration of O*_v_* in the sample at 800 rpm (63.6%) has substantially increased compared to that in the sample at 0 rpm (53.9%).

The hydrogen temperature-programmed reduction (H_2_-TPR) results showed that in the 800 rpm sample (Figure 2d), the peaks associated with Mn^3+^ emerged at lower temperatures, suggesting an early reduction of Mn^3+^ to Mn^2+^ with only 0.76 times H_2_ utilization. The peaks observed in the oxygen temperature-programmed desorption (O_2_-TPD) spectra (Figure 2e) could be assigned to several species: O_2_ (_ads_), O^2–^, O^–^, surface O_latt_^2–^, and bulk O_latt_^2–^. These peaks were detected at temperatures of 260.8, 463.5, 563.5, 680.1, and 728.9 °C, respectively. Activation of C and O bonds significantly increased the concentration of O^–^ and led to a displacement of the surface O_latt_^2–^ and bulk O_latt_^2–^ curves toward lower temperatures, as well as a substantial increase in the O_2_ desorption (1.84 times) of the NCM-C materials.

The X-ray absorption near edge structure (XANES) analysis (Figure 2f) revealed two distinct peaks, namely, *e*_*g*_ and *t*_2*g*_, in the pre-edge region. These peaks are formed by allowing electric dipoles in the O 2p band to transition to hole states.^48^ The pre-edge peak of the 800 rpm sample exhibited a notable increase in the intensity, suggesting a rise in the O 2p hole states (∼O^2–^ → O^2–^ + δ, where δ is directly proportional to the covalent bond type).^49^ The peak of σ* is caused by the Co–O interaction in the common octahedral layer. The peak intensity of the sample after ball milling significantly decreased, indicating a weakening of Co–O in the octahedral layer.

Across the boundary area between NCM and C mixed materials in Figure 2g, at a nanoscale of 2 nm, a ring-shaped structure is observed to be composed of carbon atoms arranged in single or multiple layers (Figure 2h). This structure exhibits a stable hexagonal honeycomb architecture. Hexagonal carbon rings experience bending and distortion, providing evidence of bond activation of the NCM-C mixed materials. Figure 2i depicts the high-resolution spectra for the C 1s region, and four deconvoluted peaks were fitted in the spectra, representing carbon species: C=C (sp^2^), “defect peak” (sp^3^), C–O, and CO_3_^2–^. The sp^2^/sp^3^ ratio exhibited a drop from 1.0 (0 rpm) to 0.74 (800 rpm), suggesting an increase in the carbon defects and impaired edges of the NCM-C mixed materials. The Raman spectra (Figure 2j) exhibit two discrete peaks at 1348 and 1570 cm^–1^, corresponding to the D and G peaks of graphite, respectively. The *I*_D_/*I*_G_ value of graphite decreased from 0.72 to 0.36, implying the transformation of graphite from a multilayer to single-layer structure and an increased degree of graphitization. The peak at 2698 cm^–1^ belongs to the 2D peak of carbon, which can illustrate that the graphite layer transforms from multiple layers to a single layer.^50^

The XANES analysis of the C K-edge (Figure 2k) revealed a distinct peak at 284.4 eV, which is identified because of the splitting of the peak due to the sp^2^ hybridization of C=C bonds. Following the C and O bond activation, the concentration of isolated C=C bonds was elevated, suggesting improved interactions between transition metals and graphite that enhanced the intensity of the split peak. The increased intensity of the signal at 285.3 eV, corresponding to carbon rings with sp^2^ hybridization, indicates a higher degree of graphitization in the carbon. The decreased intensity of the signal at 293.1 eV, corresponding to carbon atoms bonded in a sp^3^ hybridized state,^51^ suggests that the degree of graphitization of carbon increased. In addition, the presence of the Rydberg peak at 290.3 eV suggests a higher level of carbon disorder.^52^ The TG-DTG results confirm the continuous weight loss of the graphite electrode, reflecting its increased internal energy and enhanced reaction activity (Figure 2l).

### 3.3 Computational Chemistry of C and O Bond Activation

**Figure 3:**
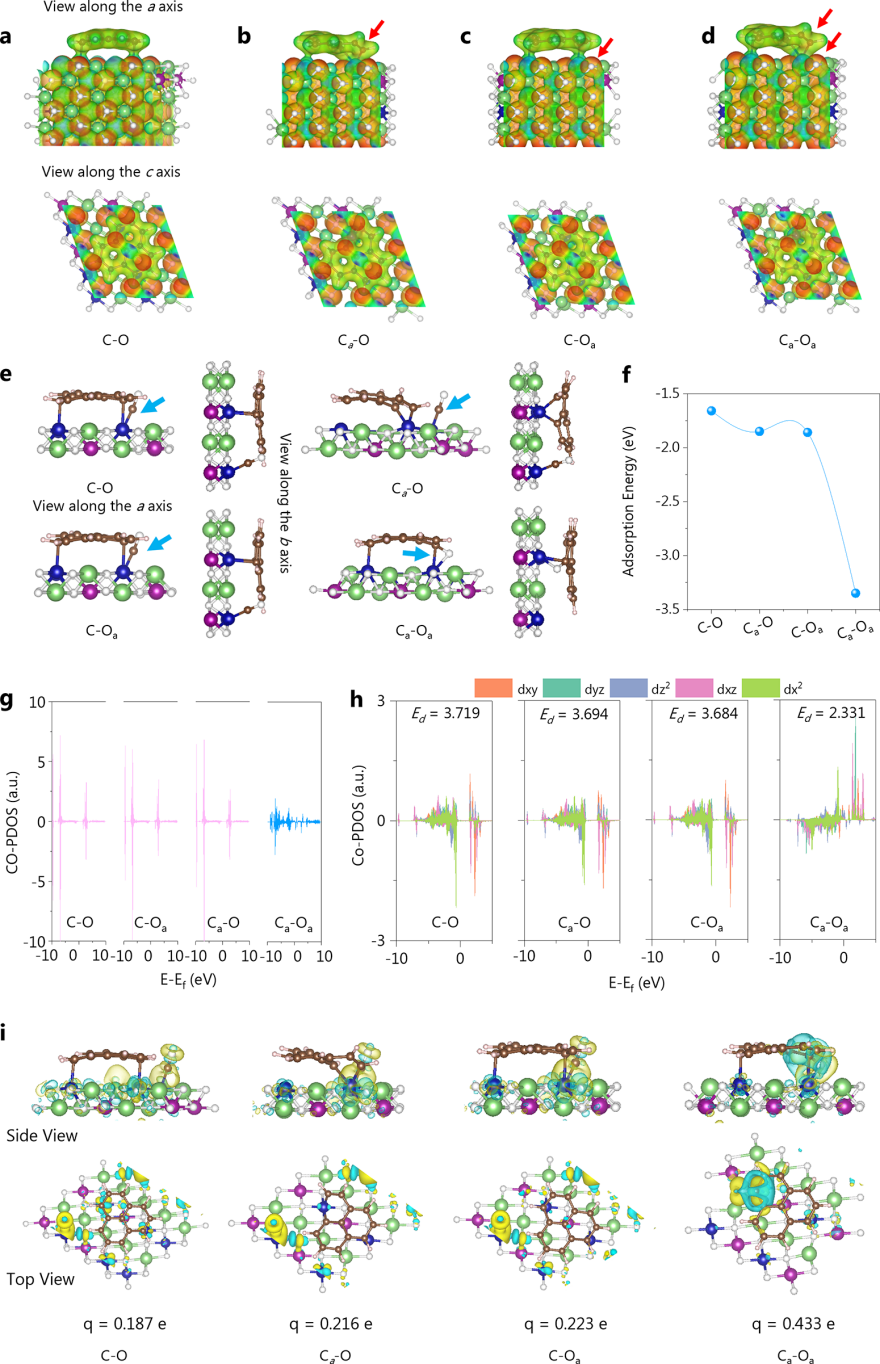
Surface maps of electrostatic potentials with different electronic structures: (a) C–O, (b) C_*a*_*–*O, (c) C–O_*a*_, and (d) C_*a*_–O_*a*_; interactions between different electron configurations (C–O, C_*a*_–O, C–O_*a*_, and C_*a*_–O_*a*_) and CO: (e) CO adsorption structure, (f) CO adsorption energy, (g) PDOS results of CO adsorption, (h) PDOS results of Co (Co as an adsorption site for CO species), and (i) results of differential charge and Bader charge; the yellow area represents the area of electron concentration, and the blue area represents the area of electron loss (green = Li, brown = C, white = O, purple = Mn, blue = Co, and gray = Ni).

We created four electronic configurations to analyze the C and O bond activation using computational chemistry, namely, C–O (no activation), C_*a*_–O (C bond activation), C–O_*a*_ (O bond activation), and C_*a*_–O_*a*_ (C and O bond activation). According to the electrostatic potential (Figure 3a–d), the activation of the C bond results in an uneven distribution of potential and increases the electron density between graphite and NCM. The activation of the O bond affects the potential of nearby Co atoms. Following the synchronous activation of C and O bonds, the NCM-C activity increases because C defect sites are more likely to interact with CO and the activation of the O bond promotes the activity of Co atoms and further increases the adsorption of CO. In Figure 3e, the adsorption energies of CO for four electrical configurations were computed. Compared with C–O (−1.66 eV), C_*a*_–O (−1.85 eV), and C–O_*a*_ (−1.86 eV), synchronous activation of C and the O bond has the highest adsorption energy for CO (−3.35 eV) (Figure 3f). In the structures of C–O, C_*a*_–O, and C–O_*a*_, Co is the transition metal atom site in the NCM that adsorbs CO. In the electronic configuration of C_*a*_–O_*a*_, the C atom in CO readily adheres to the reactive carbon defect in the graphite, while the O atom easily adheres to the oxygen vacancy site of NCM (Figure 3e). Thus, the synergistic activation of the C and O bonds resulted in the highest adsorption energy for CO.

Based on the PDOS model (Figure 3g), it is evident that when CO acquires a significant number of electrons, numerous occupied states emerge near the Fermi level. This phenomenon occurs because of the introduction of electrons into the CO through the substrate after C and O bond activation. This electron injection activates CO, causing an increase in bond length and leading to significant adsorption of CO onto the substrate. The synchronous activation of C and O bonds would cause the d-band center of active site Co to shift upward and closer to the Fermi level, improving the adsorption performance of CO on the reduction intermediate at the Co site and thus accelerating the reaction kinetics of the rate-determining step, according to our calculations of the PDOS results based on Co (Figure 3h).

We computed the Bader and differential charge transfer of four electronic structures in the interaction with CO, as shown in Figure 3i. The Bader charge results confirm that the activated bond model has the highest charge accumulation and consumption, confirming that the synchronous activation of the C and the O bonds can lead to more accurate electron transfer. The CO has a total of 10 valence electrons. After adsorption, the CO valence electrons in the four models exhibit values of 10.187, 10.216, 10.223, and 10.433 |e|. This indicates that the charge transfer between C_*a*_–O_*a*_ and CO is the most significant, with a value of 0.433 e. The CO becomes activated upon electron transfer, resulting in an elongation of the C–O bond length. Following the adsorption of CO in the four models, the bond lengths of CO are measured to be 1.1680, 1.1705, 1.1708, and 1.3206 Å. These measurements align with the results of Bader charge analysis, suggesting the highest level of adsorption activity for the oxidatively charged carbons for CO.

### 3.4 Preferential Decomposition of NCM and Environmental Benefit Analysis

**Figure 4:**
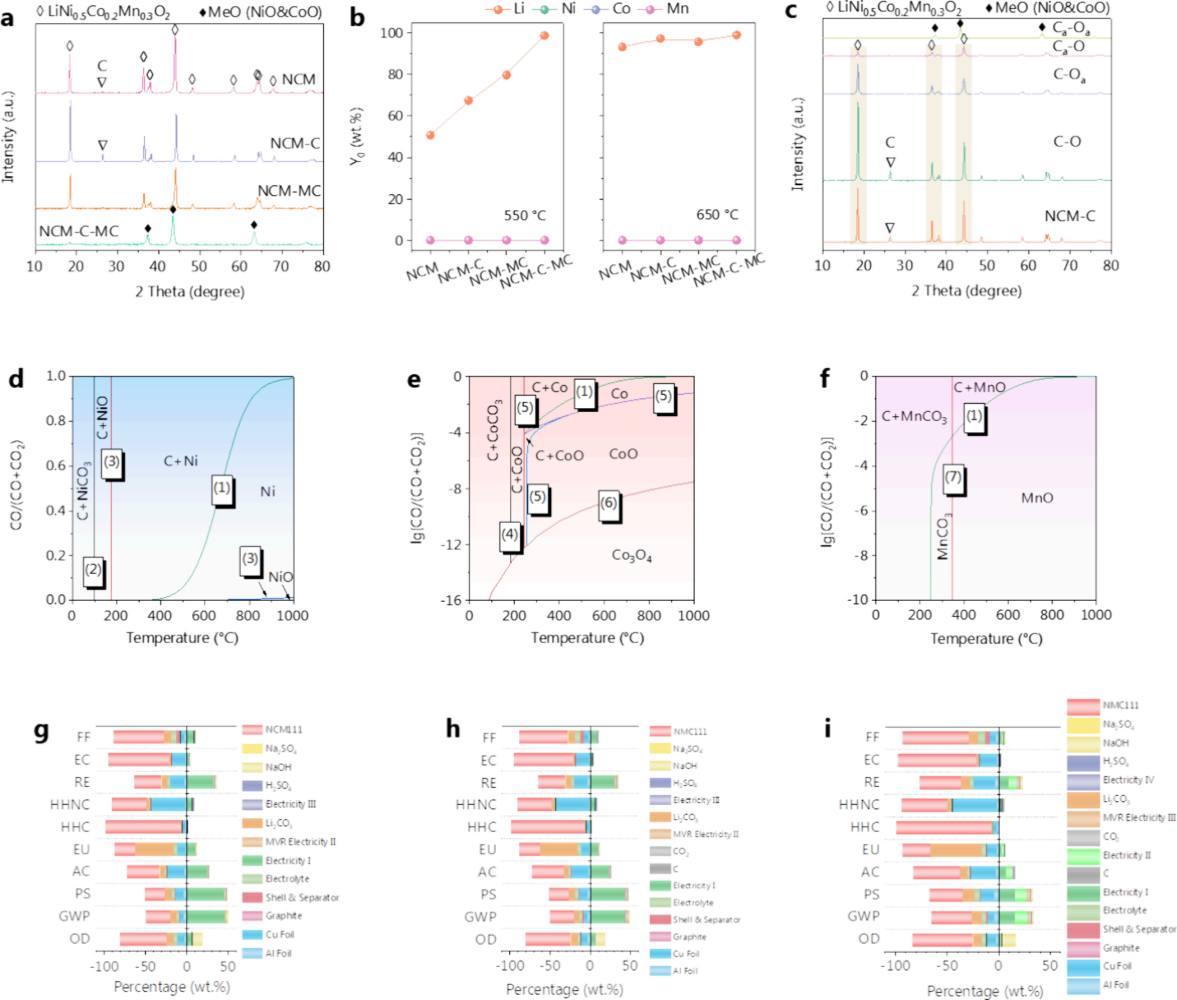
(a) XRD patterns of different NCM-C mixed materials, (b) percentage of Li released from different NCM-C mixed materials (after 550 and 650 °C pyrolysis), (c) XRD patterns of different NCM-C mixed materials after pyrolysis, Boudouard reaction, and thermodynamic equilibrium diagrams of transition metal–C–O: (d) Ni, (e) Co, and (f) Mn (the green line represents the Boudouard reaction), the percentage contribution of different materials and energy consumption to the recovery of NCM cathode materials: (g) direct pyrolysis (Route I), (h) carbothermal reduction processing (Route II), and (i) C_*a*_–O_*a*_ processing (Route III).

Various NCM and NCM-C samples (Tables S3 and S4) were used to investigate their specific phase transition during pyrolysis recycling. During the control tests, NCM, NCM-MC, and NCM-C preserved the crystal structures of LiNi_0.5_Co_0.2_Mn_0.3_O_2_ even after the pyrolysis process. In contrast, only NCM-C-MC exhibited NiO and CoO phases under identical processing conditions (Figure 4a). The XRD patterns at different pyrolysis temperatures confirmed that the deoxidation of NiO in NCM-C-MC occurred earlier at 550 and 650 °C (Figure S9), enhancing the selective phase change of NCM and increasing the efficiency of Li release (Figure S10a). Compared with NCM, NCM-MC, and NCM-C, the NCM-C-MC sample demonstrated a clear advantage and achieved an accelerated and complete release of Li at 550 °C (Figure 4b).

In further analysis, the combinations of C–O, C_*a*_–O, and C–O_*a*_ preserved the crystal structures of LiNi_0.5_Co_0.2_Mn_0.3_O_2_. In contrast, C_*a*_–O_*a*_ exhibited a preference for transforming into a mixed phase of NiO/CoO (Figure 4c). After 450 °C pyrolysis, C_*a*_–O_*a*_ exhibited the highest Li release efficiency (62.5 wt %) compared to C–O (10.1 wt %), C_*a*_–O (12.1 wt %), and C–O_*a*_ (32.3 wt %) (Figure S10b). This confirms that the activation of C and O bonds causes selective changes in its crystal structures, affording easier transition from a spinel structure to transition metal oxide and resulting in a low-temperature release of Li. Linear regression analysis, with an *R*^2^ value of 0.97 and the equation *y* = 0.06*x* + 17.3, established a direct proportional correlation between the efficiency of Li release and the extent of C and O bond activation (Figure S10c,d). These results confirm that the activation of the C and the O bonds can significantly accelerate the release efficiency of Li.

As CO is the main active species for the gasification reduction of LiNi_0.5_Co_0.2_Mn_0.3_O_2_/graphite, the influences of temperature and energy storage were analyzed on the equilibrium constants and equilibrium CO pressure fractions of carbon gasification reactions. When energy storage, total pressure, and inert gas partial pressure are constant, the equilibrium CO pressure fraction increases with temperature (Figure S11a). When temperature, total pressure, and inert gas partial pressure are constant, the equilibrium CO pressure fraction increases with energy storage (Figure S11b). To study the equilibrium state of the multiphase reaction system after graphite gasification to produce CO and CO_2_, the thermodynamic equilibrium diagrams of CO reduction of transition metal oxides (Table S5) were overlaid with the Boudouard curve at *P* = *P*^θ^ to obtain the thermodynamic phase equilibrium diagrams as shown in Figure 4d (C–O–Ni), Figure 4e (C–O–Co), and Figure 4f (C–O–Mn). The Boudouard reaction curve would shift to the right with increasing temperature, leading to early and accelerated excitation of the reduction of Ni, Co, and Mn.^53^ The increase in CO partial pressure of the CO would lead to a faster conversion of NiO to Ni (eqs 1–3, Table S5). The Co species would undergo a phase transition from Co_3_O_4_ → CoO → Co (eqs 4–6, Table S5). The Mn phase is relatively stable and can only exist in the form of MnO (eq 7, Table S5).

Possible equations at a reaction temperature of 650 °C can be described as follows:
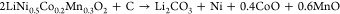
II

It has been recognized that the temperature of pyrometallurgical processing is generally above 1000 °C, which significantly reduces the economic and environmental merits of retired lithium battery recycling.^54^ We performed LCA to demonstrate the environmental benefits of the proposed C and O bond activation route (Route III; reaction temperature of 450 °C) over conventional methods (direct pyrolysis, Route I: reaction temperature of 800 °C, and carbothermal reduction, Route II: reaction temperature of 650 °C) for recycling the retired LIB cathode materials (Figure S12). LCA data inventory is provided in Table S6, and the data sources are provided in Tables S7 and S8. The percentage contributions of various materials and electricity to the environmental impact are displayed in Figure 4g–i. Compared with Routes I and II, Route III can mitigate the negative environmental impact. For example, in the GWP and PS indicators, Routes I and II have a proportion closer to 50%, while in Route III, this value is much smaller than 50%. While the use of chemicals and electricity impose detrimental effects on the environment, recycling of NCM111, Li_2_CO_3_, Al foil, and Cu foil of retired LIBs can generally make a beneficial contribution to the global environmental impact. The carbon footprint evaluation shows that recycling 1.0 kg of spent LIBs via Route I, II, and III has global warming potentials of 0.23, 0.11, and −4.57 kg CO_2_ eq, respectively. Furthermore, the PS results of the three routes are −7.4 × 10^–3^, −3.7 × 10^–2^, and −3.6 × 10^–1^ kg O_3_ eq, respectively, confirming that Route III also has a significant contribution to the reduction of smog. These results demonstrate that the C and O bond activation induced by mechanochemical processing can greatly reduce the associated CO_2_ emissions and significantly benefit the global environmental system for the sake of retired LIB recycling.^55−57^

## 4 Environmental Implications

High carbon emissions and energy consumption during the pyrolysis recycling process of retired LIBs hinder the global environmental outlook of the electric vehicle industry. We developed a low-temperature pyrolysis technology for NCM cathode materials that significantly reduces carbon emissions during the recycling process. Our findings demonstrate that the synchronous activation of C bond of graphite electrode and O bond in NCM oxide cathode electrode (LiNi_0.5_Co_0.2_Mn_0.3_O_2_) together can induce the formation of a graphite-spinel oxide structure in the NCM-C mixed materials. This process enhances the internal energy storage of carbon materials and spinel structure oxides and facilitates charge transfer. These contribute to the shifts in the gas–solid equilibrium within the multiphase system, dominated by the Boudouard reaction. Consequently, this accelerates the preferred phase transition of NCM cathode materials and the rapid release of Li at a low temperature of 550 °C for 10 min, thereby reducing emissions of pollutants (e.g., RE, AC, PS, and OD) and greenhouse gases (4570 kg CO_2_ emissions per ton) in the retired LIB recycling system. Overall, this work advances our mechanistic understanding of how a low-carbon strategy can reduce the decomposition temperature of NCM active crystals, thus mitigating the associated carbon footprints and fostering the sustainable development of the electric vehicle industry. Our proposed route can be applied to scalable engineering applications for the recycling of retired LIBs worldwide and demonstrates enormous potential for energy saving prospects.

## Supporting Information Available

HR-TEM results of NCM samples (Figure S1); particle size distribution of NCM and C samples (Figure S2); SEM-EDS mapping results of NCM samples (Figure S3); XPS spectra of NCM-C mixed material before and after C and O bond activation (Figure S4); high-resolution XPS spectra of different elements before and after C and O bond activation: (a) Ni 2p, (b) Mn 2p, (c) Co 2p, (d) O 1s, (e) F 1s, and (f) Li 1s (Figure S5); DSC fitting of (a) C, (b) NCM, and (c) NCM-C samples (Figure S6); (a) XRD patterns, (b) TG-DTG curve of NCM before and after C and O bond activation, and (c) XRD patterns of different samples (Figure S7); (a) EPR and (b) XPS analysis of O 1s sample before and after C and O bond activation (Figure S8); XRD patterns of NCM-C mixed materials (a) 0 rpm and (b) 800 rpm after pyrolysis at different temperatures (Figure S9); (a) percentage of Li released from different NCM-C samples, (b) percentage of Li released from different NCM-C mixed materials, (c) release efficiency of Li from NCM-C mixed materials at different activation speeds, and (d) linear fitting curve between efficiency of Li release and extent of bond activation (Figure S10); (a) influence of temperature on the equilibrium constant and equilibrium CO pressure fraction of carbon gasification reaction (Δ*G* = 0, *a* = 1, *b* = 0), (b) influence of energy storage on the equilibrium constant and equilibrium CO pressure fraction of carbon gasification reaction (*T* = 600 °C, *a* = 1, *b* = 0) (Figure S11); (a) various technical routes for sustainable recovery of NCM cathode materials (Route 1: direct pyrolysis, Route 2: carbothermal reduction processing, and Route 3: C*_a_*–O*_a_* reduction processing), (b) quantitative comparison of different LCA parameters in the recovery routes of NCM cathode materials, the percentage contribution of different materials and energy on (c) GWP and (d) PS (Figure S12); weight proportion of each 1.0 kg spent Li(Ni_0.5_Co_0.2_Mn_0.3_)O_2_ battery (Table S1); elemental proportion in spent Li(Ni_0.5_Co_0.2_Mn_0.3_)O_2_ cathode material (Table S2); different NCM experimental samples (Table S3); different NCM experimental samples (Table S4); gasification reactions of C–O–Li/Ni/Co/Mn (Table S5); LCA data inventory for recycling 1.0 kg spent Li(Ni_0.5_Co_0.2_Mn_0.3_)O_2_ battery via different routes (Table S6); LCA data inventory and source for NCM111 hydroxide (Table S7); LCA data source for spent Li(Ni_0.5_Co_0.2_Mn_0.3_)O_2_ battery recycling by different routes (Table S8); digestion method (Note S1); characterization methods (Note S2); calculation details of LCA (Note S3) (PDF)
