# Supplementary material for: Activating Carbon and Oxygen Bonds for Low-Temperature Thermal Decomposition of Spent Lithium-Ion Battery Cathode Materials
Source: Environ Sci Technol. 2025 Mar 6;59(10):5348–58. doi: 10.1021/acs.est.4c12200 (PMC11924225; doi:10.1021/acs.est.4c12200)
Supplement: Supplementary file 1 — es4c12200_si_001.pdf [file es4c12200_si_001.pdf]

Activating Carbon and Oxygen Bonds for Low-temperature Thermal Decomposition of Spent Lithium-ion Battery Cathode Materials

Kang Liu [a], Xiaohong Zhu [b, c], Yuying Zhang [a, c], Mengmeng Wang [a], Roya Maboudian [c], Daniel S. Alessi [d], Daniel C.W. Tsang [a, *]

[a] Department of Civil and Environmental Engineering, The Hong Kong University of Science and Technology, Clear Water Bay, Hong Kong 999077, China

[b] Department of Civil and Environmental Engineering, University of California Berkeley, Berkeley, California 94720, United States

[c] Department of Chemical and Biomolecular Engineering, University of California Berkeley, Berkeley, California 94720, United States

[d] Department of Earth and Atmospheric Sciences, University of Alberta, Edmonton, Alberta T6G 2E3, Canada

*** Corresponding author:** D.C.W. Tsang

E-mail address: cedan@ust.hk

36 Pages, 11 Figures, 8 Tables, and 3 Notes

Table of Contents

Figure S1. HR-TEM results of NCM samples (0 rpm up and 800 rpm down). 3
Figure S2. Particle size distribution of NCM and C samples.. 4
Figure S3. SEM-EDS mapping results of NCM samples (0 rpm up and 800 rpm down)............................... 5
Figure S4. XPS spectra of NCM-C mixed material before and after C and O bond activation....................... 6
Figure S5. High-resolution XPS spectra of different elements before and after C and O bond activation: (a) Ni *2p*, (b) Mn *2p*, (c) Co *2p*, (d) O *1s*, (e) F *1s*, and (f) Li *1s*... 7
Figure S6. DSC fitting of (a) C, (b) NCM, and (c) NCM-C samples (0 rpm up and 800 rpm down)................ 8
Figure S7. (a) XRD patterns (NCM is the original sample, NCM-MC is processed by C and O bond activation at 800 rpm, and NCM-MC-450 is NCM-MC pyrolyzed at 450 °C), (b) TG-DTG curve of NCM before and after C and O bond activation (0 rpm and 800 rpm); (c) XRD patterns of different samples (C is the original sample, C-MC is processed by bond activation at 800 rpm, and C-MC-450 is C-MC pyrolyzed at 450 °C).. 9
Figure S8. (a) EPR and (b) XPS analysis of O*1s* sample before and after C and O bond activation..............10
Figure S9. XRD patterns of NCM-C mixed materials (a) 0 rpm and (b) 800 rpm after pyrolysis at different temperatures. 11
Figure S10. (a) percentage of Li released from different NCM-C samples (corresponding to Figure 4a, The pyrolysis data was obtained at 450 °C), (b) percentage of Li released from different NCM-C mixed materials (corresponding to Figure 4c), (c) release efficiency of Li from NCM-C mixed materials at different activation speeds, and (d) linear fitting curve between efficiency of Li release and extent of bond activation (corresponding to Figure S10c) (reaction conditions: graphite addition of 5 wt.%, heating temperature of 450 °C, and activation speeds of 800 rpm, and reaction time of 12 h)..12
Figure S11. (a) influence of temperature on the equilibrium constant and equilibrium CO pressure fraction of carbon gasification reaction ($\Delta G = 0$, $a = 1$, $b = 0$), (b) influence of energy storage on the equilibrium constant and equilibrium CO pressure fraction of carbon gasification reaction ($T = 600$ °C, $a = 1$, $b = 0$)..13
Figure S12. (a) various technical routes for sustainable recovery of NCM cathode materials (Route 1: direct pyrolysis, Route 2: carbothermal reduction processing, and Route 3: C_a-O_a reduction processing), (b) quantitative comparison of different LCA parameters in the recovery routes of NCM cathode materials. The percentage contribution of different materials and energy on (c) GWP, and (d) PS...14
Table S1. Weight proportion of each 1.0 kg spent $Li(Ni_{0.5}Co_{0.2}Mn_{0.3})O_2$ battery 16
Table S2. Elemental proportion in spent $Li(Ni_{0.5}Co_{0.2}Mn_{0.3})O_2$ cathode material..17
Table S3. Different NCM experimental samples...18
Table S4. Different NCM experimental samples...19
Table S5. Gasification reactions of C-O-Li/Ni/Co/Mn...20
Table S6. LCA data inventory for recycling 1.0 kg spent $Li(Ni_{0.5}Co_{0.2}Mn_{0.3})O_2$ battery *via* different routes....21
Table S7. LCA data inventory and source for NCM111 hydroxide[1]..24
Table S8. LCA data source for spent $Li(Ni_{0.5}Co_{0.2}Mn_{0.3})O_2$ battery recycling by different routes..................26
Note S1. Digestion method...30
Note S2. Characterization methods..31
Note S3. Calculation details of LCA..32

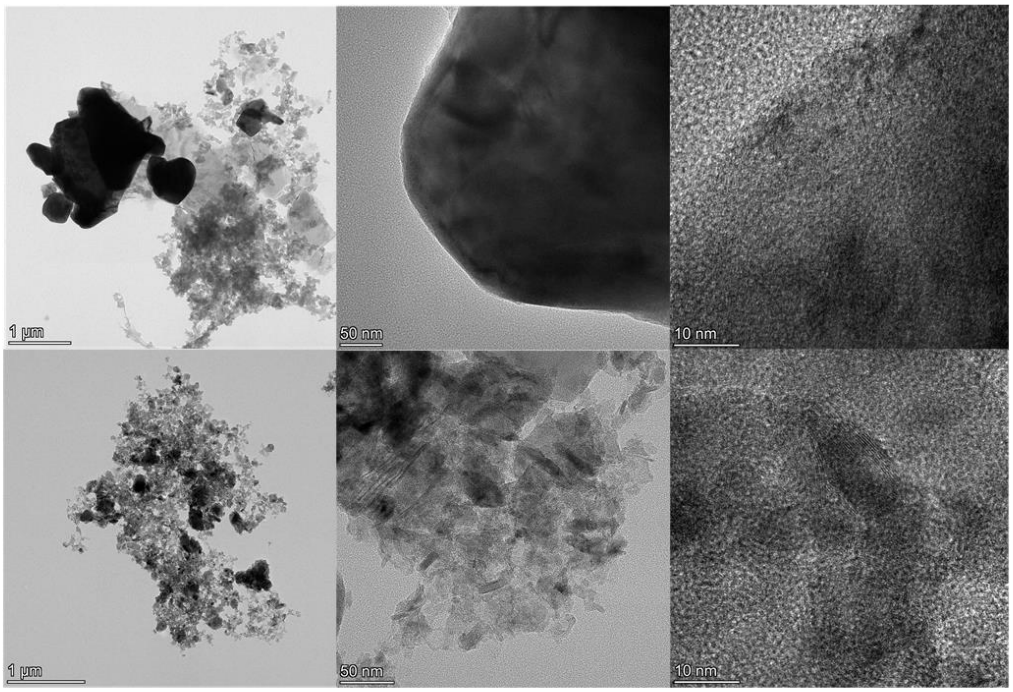

Figure S1. HR-TEM results of NCM samples (0 rpm up and 800 rpm down).

Based on the HR-TEM results, the structures of NCM and C were visible within the HR-TEM field of view prior to C and O bond activation. NCM displayed a black agglomerated structure, whereas graphite displayed a light cloud-like structure. Following the C and O bond activation, NCM particles underwent fragmentation and were subsequently combined with graphite structures. Additional structural study demonstrated that the NCM and C components created a boundary region that separated the crystalline and amorphous phases.

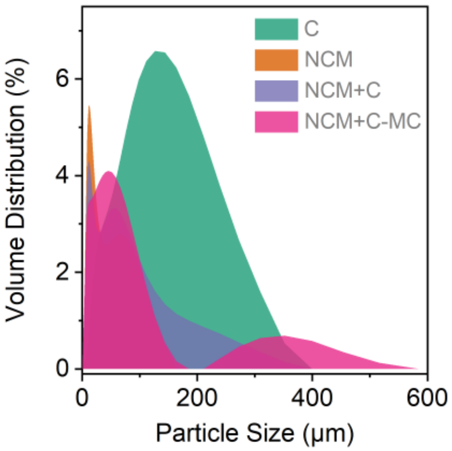

Figure S2. Particle size distribution of NCM and C samples.

According to the particle size distribution results, the particle size of graphite was 200 μm. The particle size of NCM was within the range of 20 μm. After the C and O bond activation, the particle size distribution of NCM-C material exhibits two shoulder peaks at 50 μm and 400 μm. The above results indicate that graphite is likely to form a coating on NCM.

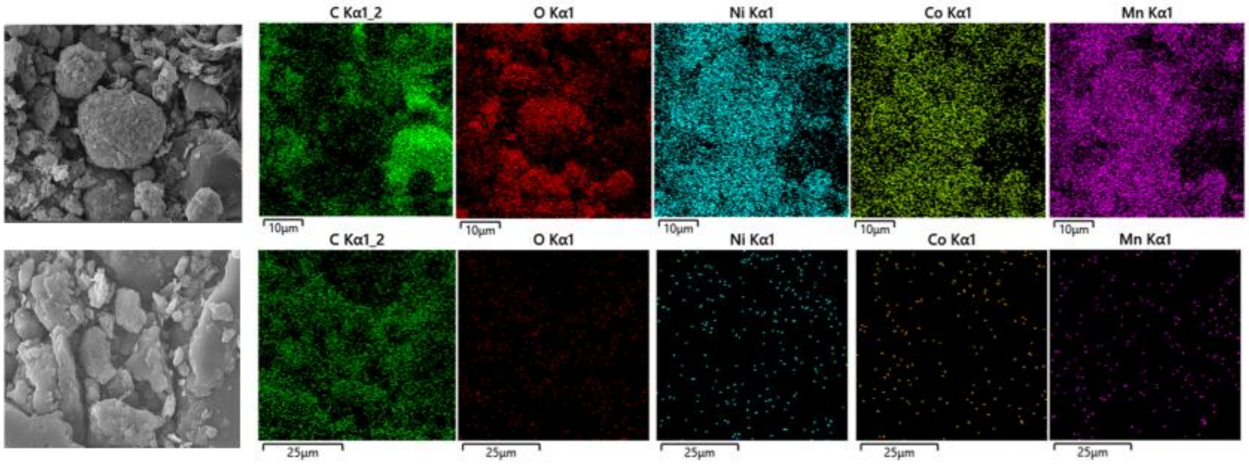

Figure S3. SEM-EDS mapping results of NCM samples (0 rpm up and 800 rpm down).

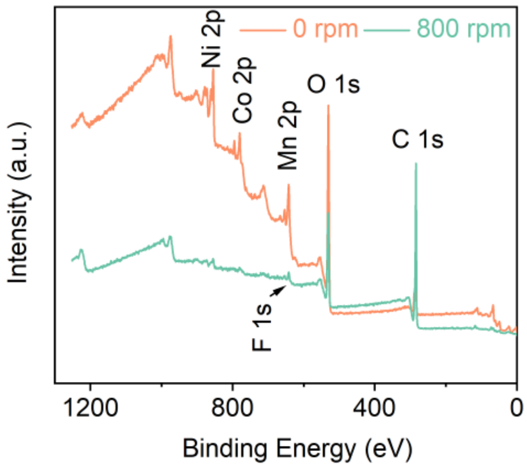

Figure S4. XPS spectra of NCM-C mixed material before and after C and O bond activation.

Compared to 0 rpm, the total peak height of C*1s* significantly increased after the C and O bond activation (800 rpm), while the total peak height of O*1s* significantly decreased.

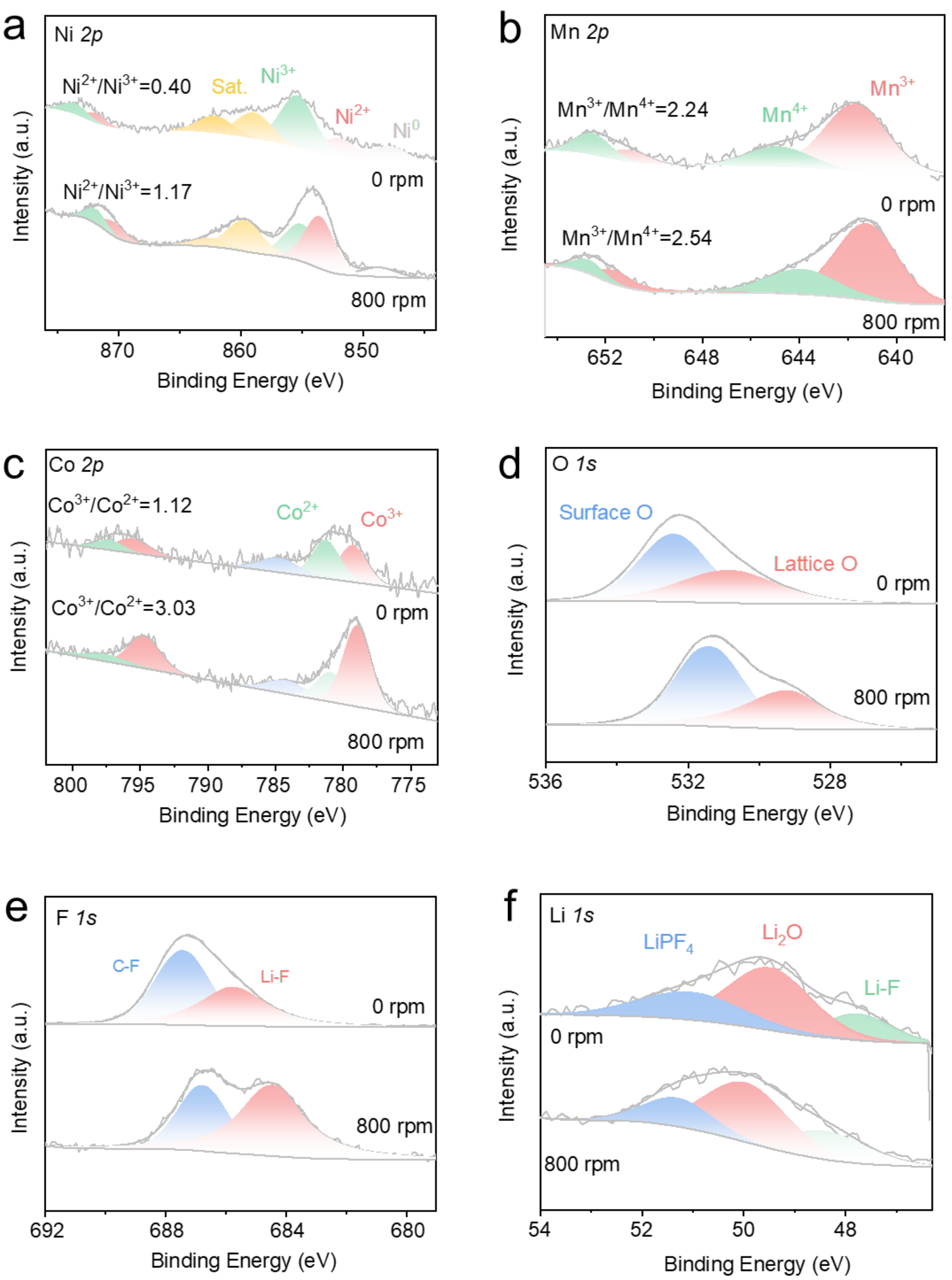

Figure S5. High-resolution XPS spectra of different elements before and after C and O bond activation: (a) Ni *2p*, (b) Mn *2p*, (c) Co *2p*, (d) O *1s*, (e) F *1s*, and (f) Li *1s*.

After the C and O bond activation, the ratio of Ni^{2+}/Ni^{3+} decreased and increased (Figure 5a), while the ratio of Mn^{3+}/Mn^{4+} increased (Figure 5b), confirming the solid-state reduction of Ni^{3+} and Mn^{4+} by mechanical force. In the remaining elemental spectra (Figures 5c-5f), we did not see any changes that could further explain the C/O element. The displacement of XPS characteristic peaks is due to the size effect of NCM-C particle size reduction. After the crystal is ground, the XPS peaks of Li, Co, and O show significant shifts, but there is no difference in peak fitting.

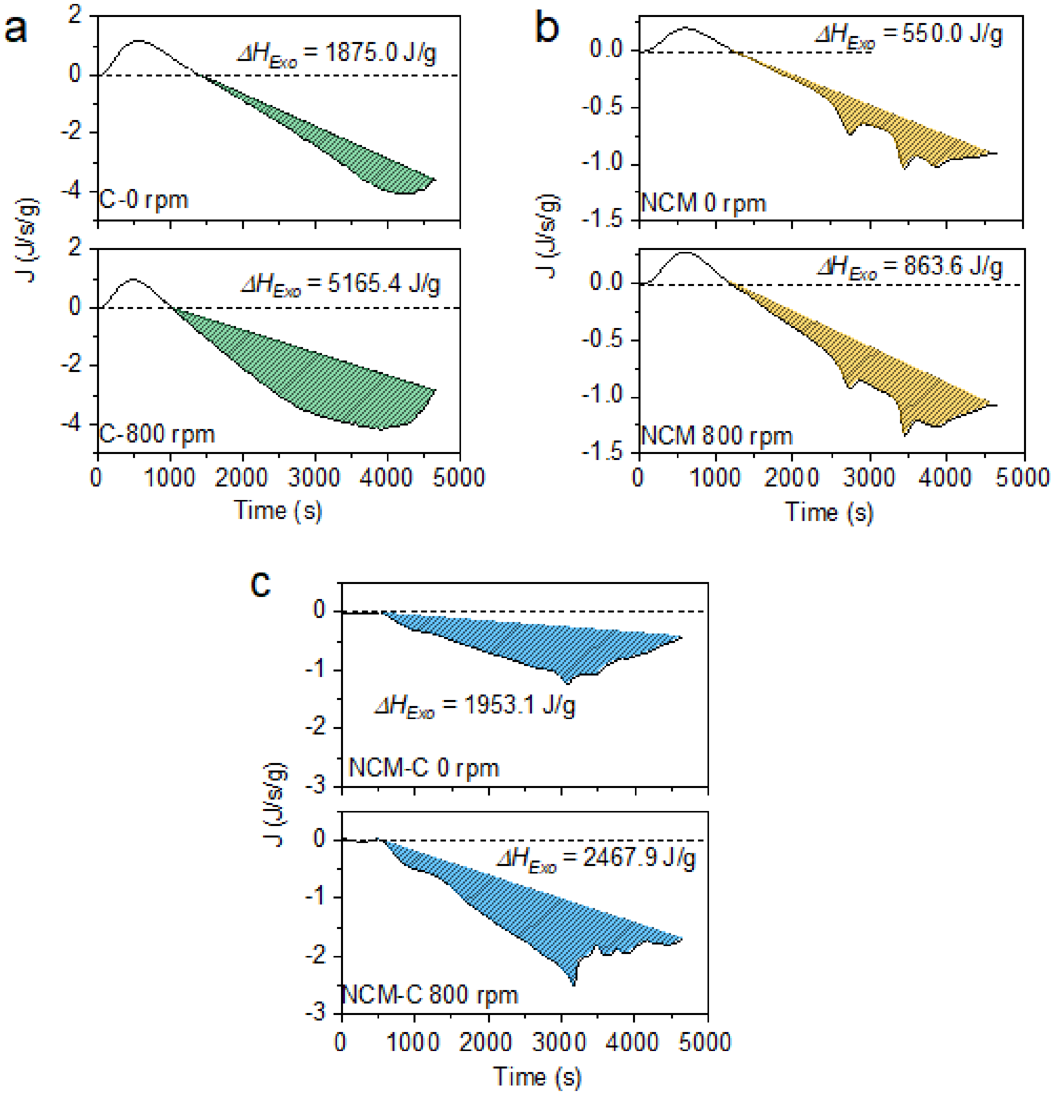

Figure S6. DSC fitting of (a) C, (b) NCM, and (c) NCM-C samples (0 rpm up and 800 rpm down).

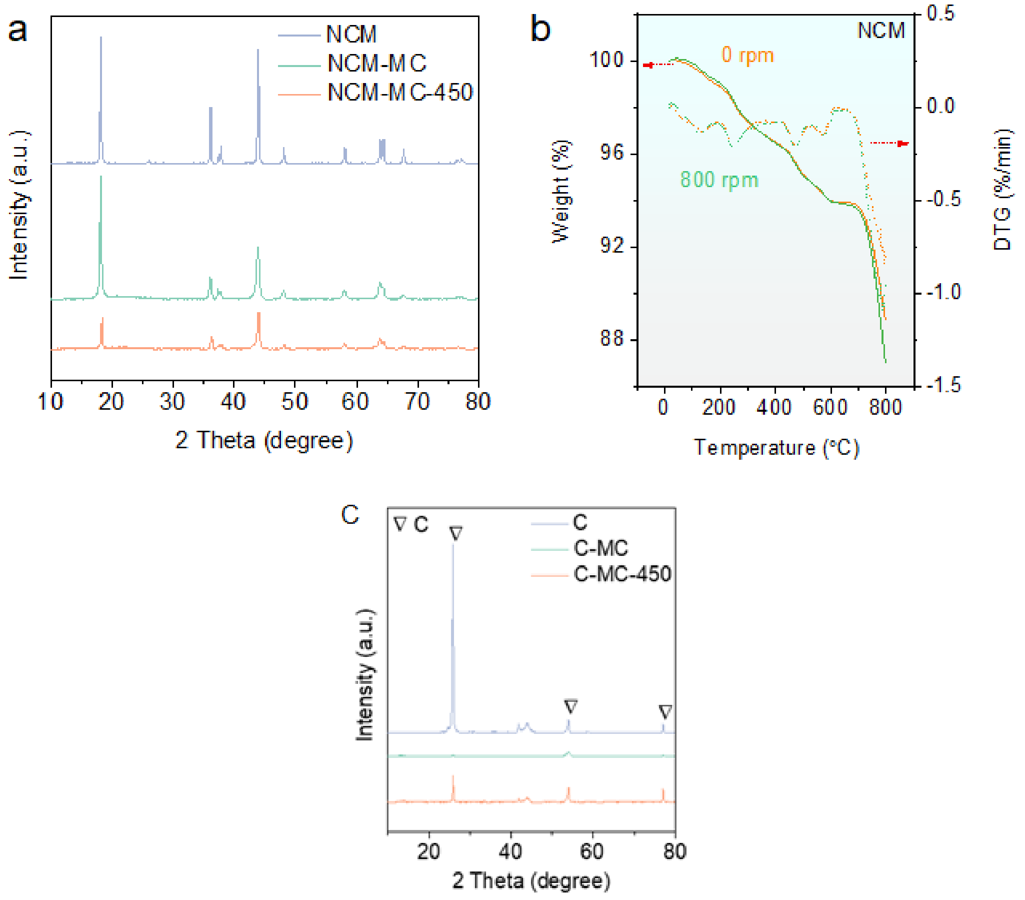

Figure S7. (a) XRD patterns (NCM is the original sample, NCM-MC is processed by C and O bond activation at 800 rpm, and NCM-MC-450 is NCM-MC pyrolyzed at 450 °C), (b) TG-DTG curve of NCM before and after C and O bond activation (0 rpm and 800 rpm); (c) XRD patterns of different samples (C is the original sample, C-MC is processed by bond activation at 800 rpm, and C-MC-450 is C-MC pyrolyzed at 450 °C).

According to Figure S7a, the diffraction peaks of the NCM sample exhibit dwarfing and broadening after C and O bond activation, confirming the refinement and distortion of the NCM crystal particles. After pyrolysis at 450 °C, the diffraction peak of the NCM-MC sample continued to dwarf, indicating the possible release of Li, and confirming the decomposition of NCM. The TG-DTG results of Figure S7b confirm that there is no significant weight loss in the NCM sample before and after C and O bond activation, confirming that the spinel structured oxide is extremely stable at high temperatures. This result is consistent with XRD pattern results. According to Figure S7c, the diffraction peaks of sample C exhibited significant dwarfing and broadening after C and O bond activation, and were close to disappearing, confirming the amorphous nature of graphite crystal particles. After pyrolysis at 450 °C, the diffraction peak of sample C partially recovered its crystal structure, confirming the release of stored internal energy during C and O bond activation. However, due to the fact that complete recovery of graphite may require higher temperatures, the diffraction peaks did not return to their original height.

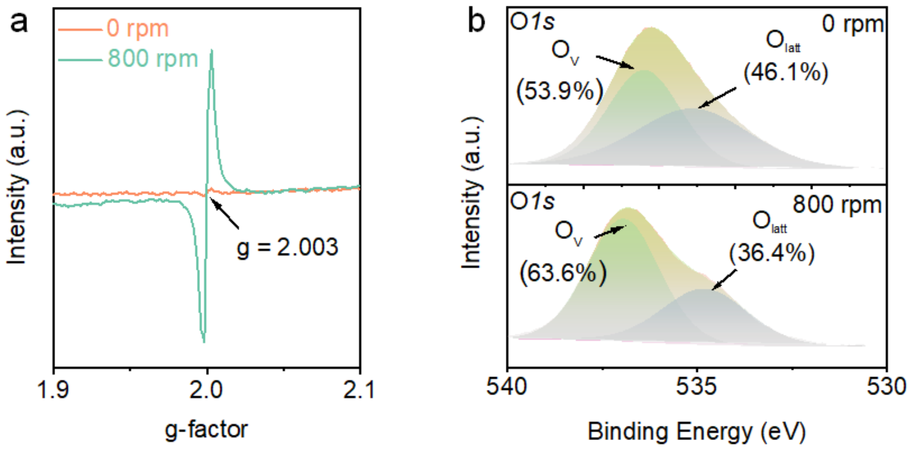

Figure S8. (a) EPR and (b) XPS analysis of O*1s* sample before and after C and O bond activation.

The electron paramagnetic resonance (EPR) results demonstrated that bond activation disrupts metal-oxygen bonds. This resulted in the emergence of a distinct peak at g = 2.004 (Figure 8a), which is associated with oxygen vacancies. XPS analysis (Figure 8b) shows that the surface concentration of O_V in the sample at 800 rpm (53.9%) has substantially increased compared to the sample at 0 rpm (63.6%).

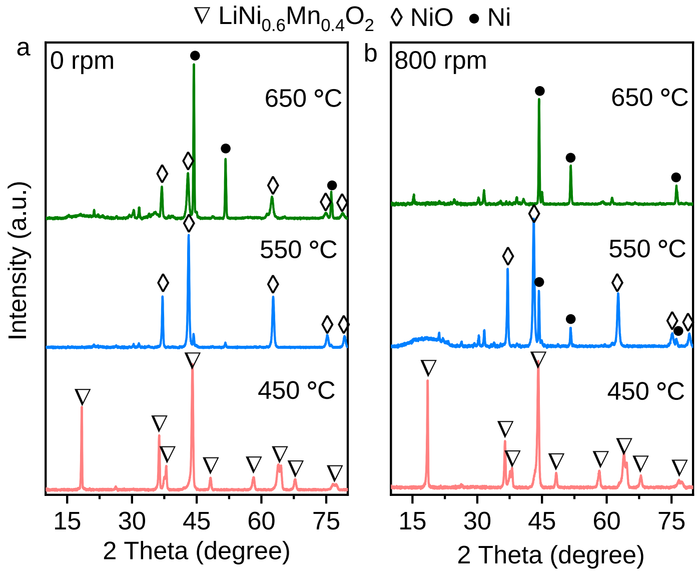

Figure S9. XRD patterns of NCM-C mixed materials (a) 0 rpm and (b) 800 rpm after pyrolysis at different temperatures.

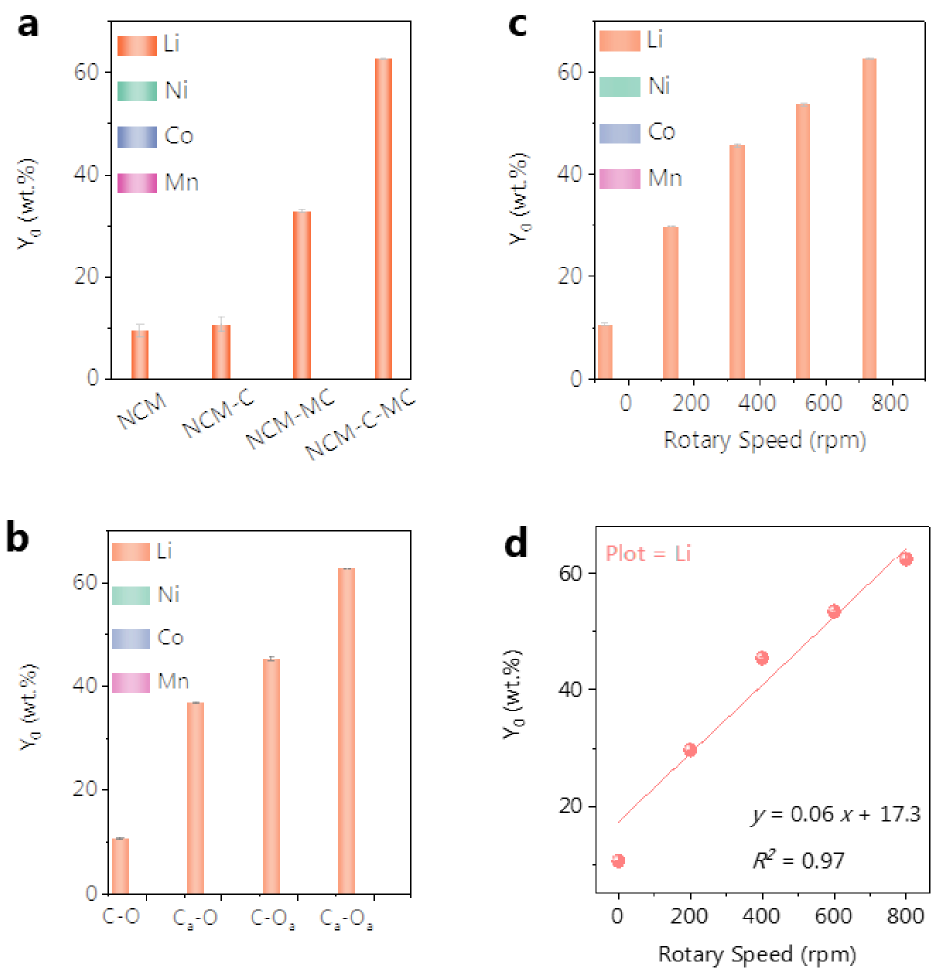

Figure S10. (a) percentage of Li released from different NCM-C samples (corresponding to Figure 4a, The pyrolysis data was obtained at 450 °C), (b) percentage of Li released from different NCM-C mixed materials (corresponding to Figure 4c), (c) release efficiency of Li from NCM-C mixed materials at different activation speeds, and (d) linear fitting curve between efficiency of Li release and extent of bond activation (corresponding to Figure S10c) (reaction conditions: graphite addition of 5 wt.%, heating temperature of 450 °C, and activation speeds of 800 rpm, and reaction time of 12 h).

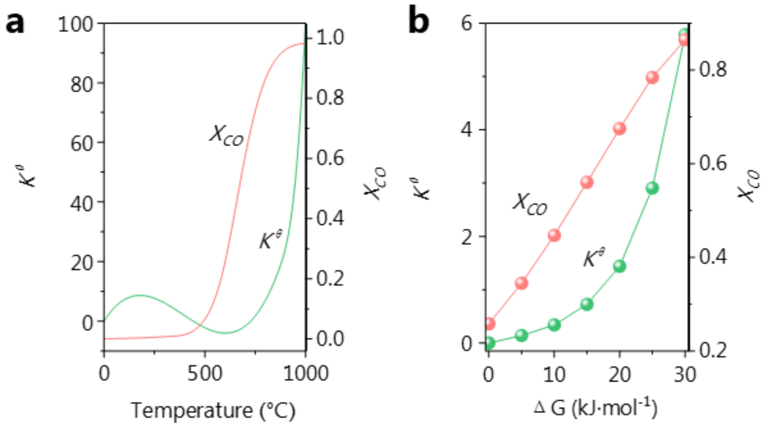

Figure S11. (a) influence of temperature on the equilibrium constant and equilibrium CO pressure fraction of carbon gasification reaction (ΔG = 0, a = 1, b = 0), (b) influence of energy storage on the equilibrium constant and equilibrium CO pressure fraction of carbon gasification reaction (T = 600 ℃, a = 1, b = 0)

Brin Chaoke, Guo Ting, Zhang Bangwen, etc Thermodynamic analysis of carbon thermal reduction reaction in mechanically activated graph-Fe_2O_3 System. Mining and Metallurgical Engineering. 2013, (4).

Brin Chaoke, Zhang Bangwen, Zhao Ruichao, etc The influence of mechanical energy storage on the thermodynamics of carbon thermal reduction of graphite magnetite system. Mining and Metallurgical Engineering. 2012, (5)

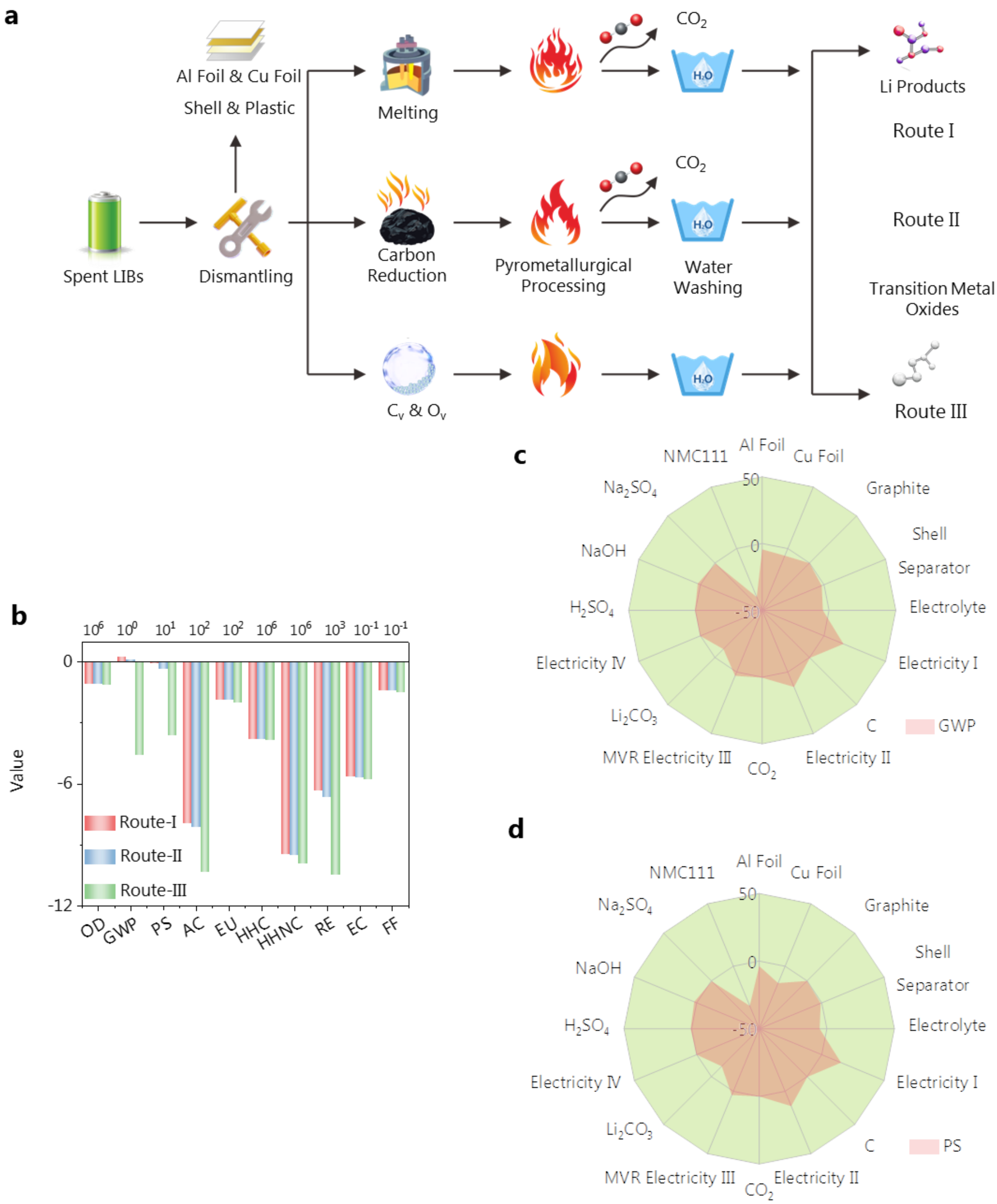

Figure S12. (a) various technical routes for sustainable recovery of NCM cathode materials (Route 1: direct pyrolysis, Route 2: carbothermal reduction processing, and Route 3: C_a-O_a reduction processing), (b) quantitative comparison of different LCA parameters in the recovery routes of NCM cathode materials. The percentage contribution of different materials and energy on (c) GWP, and (d) PS.

To demonstrate the technological benefits of the proposed C_a-O_a route over conventional methods (direct pyrolysis and carbothermal reduction processing) for recovering the retired LIB cathode materials, we performed a life cycle assessment (LCA) of the three recovery routes (Figure 11a). A total of ten indicators, including ozone depletion (OD, kg CFC-11 eq), global warming potential (GWP, kg CO_2 eq), smog (PS, kg O_3 eq), acidification (AC, mol SO_2 eq), eutrophication (EU, kg N eq), carcinogenic (HHC, CTUh), non-carcinogenic (HHNC, CTUh), respiratory effects (RE, kg PM2.5eq), ecotoxicity (EC, CTUe), and fossil fuel depletion (FF, MJ surplus), were selected to comprehensively describe the impact of the process life cycle. Figure 11b displays the quantitative findings from the LCA of three NCM recycling routes. When GWP, PS, AC, and RE indicators were evaluated, the C_a-O_a approach showed notable technological advantages over direct pyrolysis and carbothermal reduction processing. For instance, recycling 1.0 kg of spent LIBs *via* Route I, II, and III has global warming potentials of 0.23, 0.11, and -4.57 kg CO_2 eq, respectively. In Figures 11c and 11d, we examined the percentage contributions of different materials and energy sources to the GWP and PS indicators. Smog and CO_2 emissions were caused by Electricity I and II, as the key unfavorable factors. However, the production of NCM could much outweigh these drawbacks. Overall, the C_a-O_a route significantly benefits the global environmental system.

Table S1. Weight proportion of each 1.0 kg spent $Li(Ni_{0.5}Co_{0.2}Mn_{0.3})O_2$ battery

		Weight (g)	Weight Ratio (wt.%)
Cathode Electrode	Al Foil	42.8	4.28
	Cathode Materials	392.84	39.28
Anode Electrode	Cu Foil	88	8.80
	Graphite	263.9	26.39
Shell (Plastic)		4.7	0.47
Separator (Plastic)		57.7	5.77
Electrolyte		150	15
Sum		60.94	100.00

Liu K, Xu Z, Wang M, et al. Mechanisms of Thermal Decomposition in Spent NCM Lithium-Ion Battery Cathode Materials with Carbon Defects and Oxygen Vacancies. Environmental Science & Technology, 2024, 58(48): 21362-21373.

Table S2. Elemental proportion in spent $Li(Ni_{0.5}Co_{0.2}Mn_{0.3})O_2$ cathode material

Cathode Material	Element (wt.%)					
	Li	Ni	Co	Mn	O	C
$C/Li(Ni_{0.5}Co_{0.2}Mn_{0.3})O_2$	6.03	25.64	10.15	13.98	41.19	3.01

Liu K, Xu Z, Wang M, et al. Mechanisms of Thermal Decomposition in Spent NCM Lithium-Ion Battery Cathode Materials with Carbon Defects and Oxygen Vacancies[J]. Environmental Science & Technology, 2024, 58(48): 21362-21373.

Table S3. Different NCM experimental samples

	Raw Materials		Bond Activation
NCM	NCM cathode materials	None	None
NCM-C	NCM cathode materials	Graphite	None
NCM-MC	NCM cathode materials	None	Yes
NCM-C-MC	NCM cathode materials	Graphite	Yes

MC represents C and O bond activation (800 rpm and 12 h).
Processing represents high temperature thermochemical pyrolysis treatment (450 °C and 1 h). NCM is the original NCM sample, NCM-C is a mixed sample of NCM and C, NCM-MC is the sample after C and O bond activation of the original NCM sample, and NCM-C-MC is the sample after C and O bond activation of a mixed sample of NCM and C.

Table S4. Different NCM experimental samples

	Raw Materials	Bond Activation		Bond Activation
C-O	Graphite	None	NCM cathode materials	None
C_a-O	Graphite	Yes	NCM cathode materials	None
C-O_a	Graphite	None	NCM cathode materials	Yes
C_a-O_a	Graphite	Yes	NCM cathode materials	Yes

MC represents C and O bond activation treatment (800 rpm and 12 h).
Processing represents high temperature thermochemical pyrolysis treatment (450 °C and 1 h). C-O (no activation), C_a-O (carbon bond activation), C-O_a (oxygen bond activation), and C_a-O_a (synergistic activation); NCM-C represents the original sample after physical mixing, with 5 wt.% graphite added and 95 wt.% NCM).

Table S5. Gasification reactions of C-O-Li/Ni/Co/Mn

Num	Eq. n
(1)	$2CO = C + CO_2$
(2)	$NiCO_3 = NiO + CO_2$
(3)	$NiO + CO = Ni + CO_2$
(4)	$CoCO_3 = CoO + CO_2$
(5)	$CoO + CO = Co + CO_2$
(6)	$Co_3O_4 + CO = 3CoO + CO_2$
(7)	$MnCO_3 = MnO + CO_2$

Table S6. LCA data inventory for recycling 1.0 kg spent $Li(Ni_{0.5}Co_{0.2}Mn_{0.3})O_2$ battery *via* different routes

Route I					
		Unit	Corresponding LCI	Database	Notes
Input	Electricity I	kW·h	Electricity, low voltage | market group for | APOS, U	Ecoinvent 3	Energy consumption
	Electricity II	kW·h	Electricity, low voltage {CN}| market group for | APOS, U	Ecoinvent 3	Energy consumption
	H_2SO_4	g	Sulfuric acid {RoW}| market for sulfuric acid | APOS, U	Ecoinvent 3	Material consumption
	Electricity III	kW·h	Electricity, low voltage {CN}| market group for | APOS, U	Ecoinvent 3	Energy consumption
	NaOH	g	Sodium hydroxide, without water, in 50% solution state {GLO}| market for | APOS, U	Ecoinvent 3	Material consumption
Output	Al Foil	g	Aluminium collector foil, for Li-ion battery {GLO}| aluminium collector foil production, for Li-ion battery | APOS, U	Ecoinvent 3	Avoided product/ Co-product
	Cu foil	g	Copper collector foil, for Li-ion battery {GLO}| copper collector foil production, for Li-ion battery | APOS, U	Ecoinvent 3	Avoided product/ Co-product
	Shell (Plastic)	g	Battery separator {CN}| production | APOS, U	Ecoinvent 3	Avoided product/ Co-product
	Separator (Plastic)	g	Battery separator {CN}| production | APOS, U	Ecoinvent 3	Avoided product/ Co-product
	Graphite	g	Graphite {RoW}| production | APOS, U	Ecoinvent 3	Avoided product/ Co-product
	Electrolyte	g	Electrolyte, for Li-ion battery {GLO}| electrolyte production, for Li-ion battery | APOS, U	Ecoinvent 3	Avoided product/ Co-product
	Li_2CO_3	g	Lithium carbonate {CN}| lithium carbonate production, from spodumene | APOS, U	Ecoinvent 3	Avoided product/ Co-product
	Na_2SO_4	g	Sodium sulfate, anhydrite {RoW}| sodium sulfate production, from natural sources | APOS, U	Ecoinvent 3	Avoided product/ Co-product
	NMC111 Hydroxide	g	Self-modeling	Tables S6	Avoided product/ Co-product
Route II					

		Unit	Corresponding LCI	Database	Notes
Input	Electricity I	kW·h	Electricity, low voltage | market group for | APOS, U	Ecoinvent 3	Energy consumption
	C	g	Activated carbon, granular {GLO}| market for activated carbon, granular | APOS, U	Ecoinvent 3	Material consumption
	Electricity II	kW·h	Electricity, low voltage {CN}| market group for | APOS, U	Ecoinvent 3	Energy consumption
	H_2SO_4	g	Sulfuric acid {RoW}| market for sulfuric acid | APOS, U	Ecoinvent 3	Material consumption
	Electricity III	kW·h	Electricity, low voltage {CN}| market group for | APOS, U	Ecoinvent 3	Energy consumption
	NaOH	g	Sodium hydroxide, without water, in 50% solution state {GLO}| market for | APOS, U	Ecoinvent 3	Material consumption
Output	Al Foil	g	Aluminium collector foil, for Li-ion battery {GLO}| aluminium collector foil production, for Li-ion battery | APOS, U	Ecoinvent 3	Avoided product/ Co-product
	Cu foil	g	Copper collector foil, for Li-ion battery {GLO}| copper collector foil production, for Li-ion battery | APOS, U	Ecoinvent 3	Avoided product/ Co-product
	Shell (Plastic)	g	Battery separator {CN}| production | APOS, U	Ecoinvent 3	Avoided product/ Co-product
	Separator (Plastic)	g	Battery separator {CN}| production | APOS, U	Ecoinvent 3	Avoided product/ Co-product
	Graphite	g	Graphite {RoW}| production | APOS, U	Ecoinvent 3	Avoided product/ Co-product
	Electrolyte	g	Electrolyte, for Li-ion battery {GLO}| electrolyte production, for Li-ion battery | APOS, U	Ecoinvent 3	Avoided product/ Co-product
	CO_2	g	Carbon dioxide, liquid {RoW}| market for | APOS, U	Ecoinvent 3	Emission to air
	Li_2CO_3	g	Lithium carbonate {CN}| lithium carbonate production, from spodumene | APOS, U	Ecoinvent 3	Avoided product/ Co-product
	Na_2SO_4	g	Sodium sulfate, anhydrite {RoW}| sodium sulfate production, from natural sources | APOS, U	Ecoinvent 3	Avoided product/ Co-product
	NMC111 Hydroxide	g	Self-modeling	Tables S6	Avoided product/ Co-product
Route III					

		Unit	Corresponding LCI	Database	Notes
Input	Electricity I	kW·h	Electricity, low voltage | market group for | APOS, U	Ecoinvent 3	Energy consumption
	C	g	Activated carbon, granular {GLO}| market for activated carbon, granular | APOS, U	Ecoinvent 3	Material consumption
	Electricity II	kW·h	Electricity, low voltage {CN}| market group for | APOS, U	Ecoinvent 3	Energy consumption
	Electricity III	kW·h	Electricity, low voltage {CN}| market group for | APOS, U	Ecoinvent 3	Energy consumption
	H_2SO_4	g	Sulfuric acid {RoW}| market for sulfuric acid | APOS, U	Ecoinvent 3	Material consumption
	Electricity IV	kW·h	Electricity, low voltage {CN}| market group for | APOS, U	Ecoinvent 3	Energy consumption
	NaOH	g	Sodium hydroxide, without water, in 50% solution state {GLO}| market for | APOS, U	Ecoinvent 3	Material consumption
Output	Al Foil	g	Aluminium collector foil, for Li-ion battery {GLO}| aluminium collector foil production, for Li-ion battery | APOS, U	Ecoinvent 3	Avoided product/ Co-product
	Cu foil	g	Copper collector foil, for Li-ion battery {GLO}| copper collector foil production, for Li-ion battery | APOS, U	Ecoinvent 3	Avoided product/ Co-product
	Shell (Plastic)	g	Battery separator {CN}| production | APOS, U	Ecoinvent 3	Avoided product/ Co-product
	Separator (Plastic)	g	Battery separator {CN}| production | APOS, U	Ecoinvent 3	Avoided product/ Co-product
	Graphite	g	Graphite {RoW}| production | APOS, U	Ecoinvent 3	Avoided product/ Co-product
	Electrolyte	g	Electrolyte, for Li-ion battery {GLO}| electrolyte production, for Li-ion battery | APOS, U	Ecoinvent 3	Avoided product/ Co-product
	Li_2CO_3	g	Lithium carbonate {CN}| lithium carbonate production, from spodumene | APOS, U	Ecoinvent 3	Avoided product/ Co-product
	Na_2SO_4	g	Sodium sulfate, anhydrite {RoW}| sodium sulfate production, from natural sources | APOS, U	Ecoinvent 3	Avoided product/ Co-product
	NMC111 Hydroxide	g	Self-modeling	Tables S6	Avoided product/ Co-product

Table S7. LCA data inventory and source for NCM111 hydroxide[1]

LCA data inventory					
		Amount	Unit	Corresponding LCI	Database
Input	Cobalt sulphate ($CoSO_4$)	0.56	kg	GLO: market for Cobalt sulfate	Ecoinvent 3
	Manganese sulphate ($MnSO_4$)	0.55	kg	GLO: market for manganese sulfate	Ecoinvent 3
	Nickel sulphate ($NiSO_4$)	0.56	kg	GLO: market for nickel sulfate	Ecoinvent 3
	Sodium hydroxide (NaOH)	0.89	kg	RER: soda production, solvay process	Ecoinvent 3
	Chemical plant	4×10^{-10}	pcs.	RER: chemical factory construction, organics	Ecoinvent 3
	Heat	42.6	MJ	RoW: market for heat, from steam, in chemical industry	Ecoinvent 3
Output	$(Co/N/iMn)(OH)_2$	1.0	kg	Self-modeling in this table	Self-modeling in this table
	Sodium sulphate (Na_2SO_4)	1.6	kg	Sodium sulphate [Inorganic emissions to fresh water]	Ecoinvent 3

LCA data source								
Impact category	Unit	Total	$CoSO_4$	$MnSO_4$	$NiSO_4$	NaOH	Chemical factory	Heat, from steam
OD	kg CFC-11 eq	3.68E-06	2.02E-06	3.87E-08	3.91E-07	7.20E-07	4.74E-09	5.00E-07
GWP	kg CO_2 eq	2.30E+01	1.36E+01	4.40E-01	2.65E+00	1.12E+00	6.01E-02	5.11E+00
PS	kg O_3 eq	1.30E+00	8.27E-01	3.63E-02	1.89E-01	7.42E-02	4.61E-03	1.64E-01

AC	kg SO_2 eq	2.41E-01	1.49E-01	1.25E-02	5.76E-02	5.64E-03	5.68E-04	1.64E-02
EU	kg N eq	7.83E-02	5.40E-02	1.60E-03	1.22E-02	4.44E-03	4.82E-04	5.50E-03
HHC	CTUh	1.40E-05	1.13E-05	6.13E-08	2.45E-06	1.12E-07	3.75E-08	9.79E-08
HHNC	CTUh	3.80E-05	2.98E-05	2.42E-07	6.85E-06	3.93E-07	1.12E-07	6.13E-07
RE	kg $PM_{2.5}$ eq	2.75E-02	1.76E-02	1.09E-03	5.27E-03	1.58E-03	1.05E-04	1.93E-03
EC	CTUe	1.30E+03	9.92E+02	1.34E+01	2.54E+02	2.00E+01	1.00E+01	1.09E+01
FF	MJ surplus	4.12E+01	2.58E+01	6.35E-01	5.44E+00	9.02E-01	5.21E-02	8.42E+00

Due to the lack of environmental impact data for NCM523 in the LCA software, we have adopted NCM111 as a substitute, which is acceptable in the LCA evaluation methodology.

Table S8. LCA data source for spent $Li(Ni_{0.5}Co_{0.2}Mn_{0.3})O_2$ battery recycling by different routes

Route I										
Impact category	OD	GWP	PS	AC	EU	HHC	HHNC	RE	EC	FF
Unit	kg CFC-11 eq	kg CO_2 eq	kg O_3 eq	kg SO_2 eq	kg N eq	CTUh	CTUh	kg $PM_{2.5}$ eq	CTUe	MJ surplus
Total	-1.09E-06	2.34E-01	-7.41E-03	-7.97E-02	-1.89E-02	-3.82E-06	-9.49E-06	-6.36E-03	-5.67E+01	-1.40E+01
Al foil	-4.41E-08	-6.54E-01	-4.18E-02	-3.73E-03	-2.85E-04	-4.34E-08	-1.25E-07	-6.72E-04	-7.02E-01	-3.69E-01
Cu foil	-1.55E-07	-8.63E-01	-1.50E-01	-3.85E-02	-2.39E-03	-1.60E-07	-4.92E-06	-4.01E-03	-1.05E+01	-1.02E+00
Graphite	-3.92E-10	-9.59E-03	-8.21E-04	-5.82E-05	-5.14E-06	-1.45E-10	-8.78E-10	-1.83E-05	-3.70E-03	-6.18E-03
Shell & Separator	-4.40E-09	-2.68E-01	-9.07E-03	-7.75E-04	-7.33E-05	-3.63E-09	-3.92E-09	-7.71E-05	-1.82E-01	-8.04E-01
Electrolyte	-7.15E-08	-6.88E-01	-5.93E-02	-4.60E-03	-9.82E-04	-1.66E-08	-1.60E-07	-6.35E-04	-6.21E-01	-1.27E+00
Electricity I	1.19E-07	9.13E+00	6.64E-01	4.44E-02	2.73E-03	7.51E-08	8.17E-07	7.71E-03	2.20E+00	1.67E+00
MVR Electricity II	6.06E-09	4.63E-01	3.37E-02	2.25E-03	1.39E-04	3.81E-09	4.14E-08	3.91E-04	1.11E-01	8.46E-02
Li_2CO_3	-1.46E-07	-1.33E+00	-1.20E-01	-1.03E-02	-1.18E-02	-3.02E-08	-3.66E-07	-1.72E-03	-1.22E+00	-1.44E+00
Electricity III	3.61E-10	2.76E-02	2.01E-03	1.34E-04	8.26E-06	2.27E-10	2.47E-09	2.33E-05	6.64E-03	5.04E-03
H_2SO_4	6.40E-09	5.24E-02	8.06E-03	2.56E-03	6.98E-05	8.64E-09	2.11E-07	2.15E-04	5.35E-01	1.14E-01
NaOH	2.18E-07	3.40E-01	2.25E-02	1.71E-03	2.19E-04	7.57E-09	5.89E-08	4.77E-04	2.37E-01	2.74E-01
Na_2SO_4	-3.63E-09	-8.45E-02	-5.19E-03	-4.70E-04	-4.73E-05	-3.33E-09	-1.83E-08	-1.27E-04	-8.15E-02	-5.82E-02
NMC111 Hydroxide	-1.01E-06	-5.88E+00	-3.52E-01	-7.22E-02	-6.42E-03	-3.66E-06	-5.02E-06	-7.91E-03	-4.64E+01	-1.12E+01

Route II										
Impact category	OD	GWP	PS	AC	EU	HHC	HHNC	RE	EC	FF
Unit	kg CFC-11 eq	kg CO_2 eq	kg O3 eq	kg SO_2 eq	kg N eq	CTUh	CTUh	kg PM2.5 eq	CTUe	MJ surplus
Total	-1.09E-06	1.08E-01	-3.75E-02	-8.12E-02	-1.89E-02	-3.82E-06	-9.52E-06	-6.68E-03	-5.67E+01	-1.40E+01
Al foil	-4.41E-08	-6.54E-01	-4.18E-02	-3.73E-03	-2.85E-04	-4.34E-08	-1.25E-07	-6.72E-04	-7.02E-01	-3.69E-01
Cu foil	-1.55E-07	-8.63E-01	-1.50E-01	-3.85E-02	-2.39E-03	-1.60E-07	-4.92E-06	-4.01E-03	-1.05E+01	-1.02E+00
Graphite	-3.92E-10	-9.59E-03	-8.21E-04	-5.82E-05	-5.14E-06	-1.45E-10	-8.78E-10	-1.83E-05	-3.70E-03	-6.18E-03
Shell & Separator	-4.40E-09	-2.68E-01	-9.07E-03	-7.75E-04	-7.33E-05	-3.63E-09	-3.92E-09	-7.71E-05	-1.82E-01	-8.04E-01
Electrolyte	-7.15E-08	-6.88E-01	-5.93E-02	-4.60E-03	-9.82E-04	-1.66E-08	-1.60E-07	-6.35E-04	-6.21E-01	-1.27E+00
Electricity I	1.12E-07	8.54E+00	6.21E-01	4.15E-02	2.56E-03	7.03E-08	7.64E-07	7.21E-03	2.06E+00	1.56E+00
C	7.73E-09	2.09E-01	1.27E-02	1.31E-03	1.17E-04	2.44E-09	2.05E-08	1.71E-04	7.44E-02	1.19E-01
CO2	0.00E+00	2.54E-01	0.00E+00	0.00E+00	0.00E+00	0.00E+00	0.00E+00	0.00E+00	0.00E+00	0.00E+00
MVR Electricity II	6.06E-09	4.63E-01	3.37E-02	2.25E-03	1.39E-04	3.81E-09	4.14E-08	3.91E-04	1.11E-01	8.46E-02
Li2CO3	-1.46E-07	-1.33E+00	-1.20E-01	-1.03E-02	-1.18E-02	-3.02E-08	-3.66E-07	-1.72E-03	-1.22E+00	-1.44E+00
Electricity III	3.61E-10	2.76E-02	2.01E-03	1.34E-04	8.26E-06	2.27E-10	2.47E-09	2.33E-05	6.64E-03	5.04E-03
H2SO4	6.40E-09	5.24E-02	8.06E-03	2.56E-03	6.98E-05	8.64E-09	2.11E-07	2.15E-04	5.35E-01	1.14E-01
NaOH	2.18E-07	3.40E-01	2.25E-02	1.71E-03	2.19E-04	7.57E-09	5.89E-08	4.77E-04	2.37E-01	2.74E-01
Na2SO4	-3.63E-09	-8.45E-02	-5.19E-03	-4.70E-04	-4.73E-05	-3.33E-09	-1.83E-08	-1.27E-04	-8.15E-02	-5.82E-02

NMC111 Hydroxide	-1.01E-06	-5.88E+00	-3.52E-01	-7.22E-02	-6.42E-03	-3.66E-06	-5.02E-06	-7.91E-03	-4.64E+01	-1.12E+01
Route III										
Impact category	OD	GWP	PS	AC	EU	HHC	HHNC	RE	EC	FF
Unit	kg CFC-11 eq	kg CO_2 eq	kg O_3 eq	kg SO_2 eq	kg N eq	CTUh	CTUh	kg $PM_{2.5}$ eq	CTUe	MJ surplus
Total	-1.15E-06	-4.57E+00	-3.63E-01	-1.03E-01	-2.03E-02	-3.86E-06	-9.93E-06	-1.05E-02	-5.78E+01	-1.49E+01
Al foil	-4.41E-08	-6.54E-01	-4.18E-02	-3.73E-03	-2.85E-04	-4.34E-08	-1.25E-07	-6.72E-04	-7.02E-01	-3.69E-01
Cu foil	-1.55E-07	-8.63E-01	-1.50E-01	-3.85E-02	-2.39E-03	-1.60E-07	-4.92E-06	-4.01E-03	-1.05E+01	-1.02E+00
Graphite	-3.92E-10	-9.59E-03	-8.21E-04	-5.82E-05	-5.14E-06	-1.45E-10	-8.78E-10	-1.83E-05	-3.70E-03	-6.18E-03
Shell & Separator	-4.40E-09	-2.68E-01	-9.07E-03	-7.75E-04	-7.33E-05	-3.63E-09	-3.92E-09	-7.71E-05	-1.82E-01	-8.04E-01
Electrolyte	-7.15E-08	-6.88E-01	-5.93E-02	-4.60E-03	-9.82E-04	-1.66E-08	-1.60E-07	-6.35E-04	-6.21E-01	-1.27E+00
Electricity I	3.09E-08	2.36E+00	1.72E-01	1.15E-02	7.08E-04	1.94E-08	2.12E-07	2.00E-03	5.69E-01	4.32E-01
C	2.31E-09	6.25E-02	3.80E-03	3.92E-04	3.50E-05	7.30E-10	6.11E-09	5.12E-05	2.22E-02	3.56E-02
Electricity II	2.38E-08	1.82E+00	1.33E-01	8.86E-03	5.46E-04	1.50E-08	1.63E-07	1.54E-03	4.39E-01	3.33E-01
CO_2	0.00E+00	7.58E-02	0.00E+00	0.00E+00	0.00E+00	0.00E+00	0.00E+00	0.00E+00	0.00E+00	0.00E+00
MVR Electricity III	6.06E-09	4.63E-01	3.37E-02	2.25E-03	1.39E-04	3.81E-09	4.14E-08	3.91E-04	1.11E-01	8.46E-02
Li_2CO_3	-1.46E-07	-1.33E+00	-1.20E-01	-1.03E-02	-1.18E-02	-3.02E-08	-3.66E-07	-1.72E-03	-1.22E+00	-1.44E+00
Electricity IV	3.61E-10	2.76E-02	2.01E-03	1.34E-04	8.26E-06	2.27E-10	2.47E-09	2.33E-05	6.64E-03	5.04E-03
H_2SO_4	6.40E-09	5.24E-02	8.06E-03	2.56E-03	6.98E-05	8.64E-09	2.11E-07	2.15E-04	5.35E-01	1.14E-01

NaOH	2.18E-07	3.40E-01	2.25E-02	1.71E-03	2.19E-04	7.57E-09	5.89E-08	4.77E-04	2.37E-01	2.74E-01
Na_2SO_4	-3.63E-09	-8.45E-02	-5.19E-03	-4.70E-04	-4.73E-05	-3.33E-09	-1.83E-08	-1.27E-04	-8.15E-02	-5.82E-02
NMC111 Hydroxide	-1.01E-06	-5.88E+00	-3.52E-01	-7.22E-02	-6.42E-03	-3.66E-06	-5.02E-06	-7.91E-03	-4.64E+01	-1.12E+01

Note S1. Digestion method

Temperature-programmed digestion of the metal leaching solution with HNO_3 at 180 °C for 10 h was used. The digestion solution was added to a 100 mL volumetric flask containing 5% HNO_3 volume ratio once it had reached room temperature. By using ICP-OES, the concentrations of the metals in the standard solution were ascertained. Standard solution containing metal ions have concentration intervals of 0, 0.1, 0.5, 1.0, 5.0, and 10.0 mg/L. The solutions range from 0 to 10.0 mg/L. At every experimental time node, three solution samples were taken for testing and digestion; the average result was the only one shown. The standard deviation, which quantifies the dispersion of data points relative to the mean, is shown by the error bars.

Note S2. Characterization methods

The crystal structures of mixed materials were obtained by X-ray diffraction patterns using a high-intensity monochromatic Cu-Kα source (λ = 1.5218 Å) equipped X-ray diffractometer (XRD, Bruker D8A, Germany). Raman spectroscopy (RA802, Renishaw, UK) was employed to characterize the defect levels (*e.g.,* point defects, and edge defects) and graphitization degree of carbon materials under 532 nm laser excitation. X-ray photoelectron spectroscopy (XPS) using Al Kα radiation (ESCALAB 250Xi, Thermo Fisher Scientific, U.S.A.) was employed to investigate changes in the compositions of near-surface species of mixed materials. Casa XPS software was used for charge correction and peak fitting, and to calculate the valence states of critical metals (*e.g.,* Li, Ni, Co, and Mn) and group area of C (*e.g.,* C-C, C=C, C-O, C=O, and CO_3^{2-}) and O (*e.g.,* O_v, O_{latt}, and O_{ads}) species. The compositional change in C and O species on the surface of different materials was observed. Thermogravimetric analysis-differential thermal analysis-differential scanning calorimetry (TG-DTA-DSC, STA 449 F3, NETZSCH, Germany) was employed to elucidate the thermal chemical characteristics and quantify the internal energy storage of solid materials. DSC was used to quantify the enthalpy change (ΔH_{Exo}) of individual electrodes (NCM or C) and mixed NCM-C materials. We utilized the enthalpy change to construct linear or non-linear fitting models for the internal energy storage of solid materials, thereby demonstrating the energy storage differences of various materials. X-ray absorption near-edge structure (XANES, ANSTO, Australia) spectroscopy was used to chemically identify the C and O species (soft line), revealing local structural information of elements, geometric coordination, bond length, and atomic interaction mechanisms. Temperature programmed reduction in H_2 (TPR-H_2) and temperature programmed desorption (TPD-O_2) were measured by temperature programmed chemical adsorption analyzer (Micromeritics Auto Chem II 2920, U.S.A.), to determine the oxygen vacancies and surface oxygen species composition. Electron paramagnetic resonance (Bruker ELEXSYS-II E500 CW-EPR, Germany) was used to capture unpaired electrons in solid samples and detect oxygen vacancy information in NCM-C materials.

Note S3. Calculation details of LCA

LCA was used to determine whether the NCM recovery route may have a negative influence on the environment globally. SimaPro 8.5 software (PRé Sustainability, Netherlands) was used to quantify the consequences of chemical input and energy demand. The TRACI 2.1 V1.06/US 2008 evaluation procedure—a technique for evaluating and minimizing chemical and environmental impacts—was used for this purpose. For aspects of material and energy use, the databases Ecoinvent 3 and U.S. Life Cycle Inventory (USLCI) served as the main inventories in this study.

Calculation details of LCA for recycling 1.0 kg spent $Li(Ni_{0.5}Co_{0.2}Mn_{0.3})O_2$ battery

Route I

A. Energy consumption

1. Direct pyrometallurgical process

Electricity I: The total power of the atmosphere furnace used in this study was 10.0 kW (1200 °C), the amount of solid sample that can be sintered in the atmosphere furnace was about 2.5 kg each time. First, the temperature was increased from room temperature to 800 °C at a heating rate of 10°C/min (about 78 min, 1.3 h), then holding the reaction temperature at 800 °C for 1 h. The energy consumption was 10/5 × 1.3 + 10 × 800/1200 × 1 = 9.27 kW·h.

2. Dissolution separation

Electricity II: MVR evaporation method for 1 ton of water requires the power about 23-70 kW·h, and it is assumed that it is 50 kW·h here. The solubility of Li_2CO_3 at room temperature is 1.33 g/100 g of water, so it takes 9.51 kg of water to obtain 126.5 g of Li_2CO_3. The power consumption of 9.51 kg of water with MVR evaporation technology is 50 × 9.51/1000 = 0.48 kW·h.

3. Acid Leaching process

Electricity III: The power of the magnetic stirring device adopted was 0.25 kW (900 rpm). When the stirring speed was 200 rpm, the operating power of the magnetic stirring device was 0.25 × 200 ÷ 900 = 0.056 kW. The run time of the leaching reaction was set to 0.5 h. The total electricity consumption was 0.056 × 0.5 = 0.028 kW·h.

B. Chemicals consumption and obtained products

For 1.0 kg spent NCM523 battery, 392.84 g of cathode materials (3.01% C (11.7g, M = 12 g/mol), 6.03% Li (23.69 g, M = 6.94 g/mol, n = 3.413 mol), 25.64% Ni (100.72 g, M = 58.69 g/mol, n = 1.716 mol), 10.15% Co (39.87 g, M = 58.69 g/mol, n = 0.677 mol), and 13.98% Mn (54.92 g, M = 54.938 g/mol, n = 1.0 mol)) were obtained.

1. Direct pyrometallurgical process

All the data in the direct pyrometallurgical process were derived from the chemical reaction as follows:

$$LiNi_{5/10}Co_{2/10}Mn_{3/10}O_2 \rightarrow Li_2CO_3 + NiO + CoO + MnO \quad \text{(Eq.S1)}$$

(1) Li_2CO_3 (M = 73.89 g/mol): generation of Li_2CO_3 was 3.413 mol ÷ 2 × 1 = 1.71 mol, resulting in 126.5 g.
(2) NiO (M = 74.693 g/mol): generation of NiO was 1.716 mol, resulting in 128.2 g.
(3) CoO (M = 74.93 g/mol): generation of CoO was 0.677 mol, resulting in 50.7 g.
(4) MnO (M = 70.937 g/mol): generation of MnO was 1.0 mol, resulting in 70.9 g.

2. Acid Leaching process

All the data in the acid leaching process were derived from the chemical reaction as follows:

$$Ni/Co/MnO + H_2SO_4 = Ni/Co/MnSO_4 + H_2O \quad \text{(Eq.S2)}$$

(1) H_2SO_4 (M = 98.08 g/mol): consumption of H_2SO_4 was 1.716 + 0.677 + 1.0 = 3.392 mol, resulting in a weight of H_2SO_4 equal to 332.8 g.
(2) $NiSO_4$ (M = 154.76 g/mol): generation of $NiSO_4$ was 1.716 mol, resulting in 265.6 g.
(3) $CoSO_4$ (M = 154.996 g/mol): generation of $CoSO_4$ was 0.677 mol, resulting in 104.9 g.
(4) $MnSO_4$ (M = 151 g/mol): generation of $MnSO_4$ was 1.0 mol, resulting in 151 g.

3. Chemical precipitation

All the data in the chemical precipitation were derived from the chemical reaction as follows:

$$Ni/Co/MnSO_4 + 2NaOH = Ni/Co/Mn(OH)_2 + Na_2SO_4 \quad \text{(Eq.S3)}$$

(1) NaOH (M = 40 g/mol): consumption of NaOH was (1.716 + 0.677 + 1.0) × 2 = 6.784 mol, resulting in 271.4 g.
(2) $Ni(OH)_2$ (M = 92.708 g/mol): generation of $Ni(OH)_2$ was 1.716 mol, resulting in 159.1 g.
(3) $Co(OH)_2$ (M = 92.95 g/mol): generation of $Co(OH)_2$ was 0.677 mol, resulting in 62.9 g.
(4) $Mn(OH)_2$ (M = 88.952 g/mol): generation of $Co(OH)_2$ was 1.0 mol, resulting in 88.9 g.
(5) $(Ni/Co/Mn(OH)_2$: generation of $(Ni/Co/Mn(OH)_2$ was 159.1 + 62.9 + 88.9 = 311 g.
(6) Na_2SO_4 (M = 142.04 g/mol): generation of Na_2SO_4 was 1.716 + 0.677 + 1.0 = 3.392 mol, resulting in 481.9 g.

Route II

A. Energy consumption

1. Carbothermal reduction pyrometallurgical process

Electricity I: The total power of the atmosphere furnace used in this study was 10.0 kW (1200 °C), the amount of solid sample that can be sintered in the atmosphere furnace was about 2.5 kg each time. First, the temperature was increased from room temperature to 650 °C at a heating rate of 10°C/min (about 60 min, 1 h), then holding the reaction temperature at 650 °C for 1 h. The energy consumption was 10/5 × 1 + 10 × 800/1200 × 1 = 8.67 kW·h.

2. Dissolution separation

Electricity II: MVR evaporation method for 1 ton of water requires the power about 23-70 kW·h, and it is assumed that it is 50 kW·h here. The solubility of Li_2CO_3 at room temperature is 1.33 g/100 g of water, so it takes 9.51 kg of water to obtain 126.5 g of Li_2CO_3. The power consumption of 9.51 kg of water with MVR evaporation technology is 50 × 9.51/1000 = 0.48 kW·h.

3. Acid Leaching process

Electricity III: The power of the magnetic stirring device adopted was 0.25 kW (900 rpm). When the stirring speed was 200 rpm, the operating power of the magnetic stirring device was 0.25 × 200 ÷ 900 = 0.056 kW. The run time of the leaching reaction was set to 0.5 h. The total electricity consumption was 0.056 × 0.5 = 0.028 kW·h.

B. Chemicals consumption and obtained products

For 1.0 kg spent NCM523 battery, 392.84 g of cathode materials (3.01% C (11.7g, M = 12 g/mol), 6.03% Li (23.69 g, M = 6.94 g/mol, n = 3.413 mol), 25.64% Ni (100.72 g, M = 58.69 g/mol, n = 1.716 mol), 10.15% Co (39.87 g, M = 58.69 g/mol, n = 0.677 mol), and 13.98% Mn (54.92 g, M = 54.938 g/mol, n = 1.0 mol)) were obtained.

1. Carbothermal reduction pyrometallurgical process

All the data in the carbothermal reduction pyrometallurgical process were derived from the chemical reaction as follows:

$LiNi_{5/10}Co_{2/10}Mn_{3/10}O_2 + C \rightarrow Li_2CO_3 + NiO + CoO + MnO + CO_2\ (g)$ (Eq.S4)

(1) C (M = 12.01 g/mol): addition/consumption of C was 15% mass ratio of NCM523+C mixture, that was 69.3 g.

(2) CO_2 (M = 44 g/mol): emission of CO_2 was 69.3 ÷ 12.01 = 5.77 mol, resulting in 253.9 g.

(3) Li_2CO_3 (M = 73.89 g/mol): generation of Li_2CO_3 was 3.413 mol ÷ 2 × 1 = 1.71 mol, resulting in 126.5 g.

(4) NiO (M = 74.693 g/mol): generation of NiO was 1.716 mol, resulting in 128.2 g.

(5) CoO (M = 74.93 g/mol): generation of CoO was 0.677 mol, resulting in 50.7 g.

(6) MnO (M = 70.937 g/mol): generation of MnO was 1.0 mol, resulting in 70.9 g.

2. Acid Leaching process

All the data in the acid leaching process were derived from the chemical reaction as Eq.S2.

(1) H_2SO_4 (M = 98.08 g/mol): consumption of H_2SO_4 was 1.716 + 0.677 + 1.0 = 3.392 mol, resulting in 332.8 g.
(2) $NiSO_4$ (M = 154.76 g/mol): generation of $NiSO_4$ was 1.716 mol, resulting in 265.6 g.
(3) $CoSO_4$ (M = 154.996 g/mol): generation of $CoSO_4$ was 0.677 mol, resulting in 104.9 g.
(4) $MnSO_4$ (M = 151 g/mol): generation of $MnSO_4$ was 1.0 mol, resulting in 151 g.

3. Chemical precipitation

All the data in the chemical precipitation were derived from the chemical reaction described as Eq.S3.
(1) NaOH (M = 40 g/mol): consumption of NaOH was (1.716 + 0.677 + 1.0) × 2 = 6.784 mol, resulting in 271.4 g.
(2) $Ni(OH)_2$ (M = 92.708 g/mol): generation of $Ni(OH)_2$ was 1.716 mol, resulting in 159.1 g.
(3) $Co(OH)_2$ (M = 92.95 g/mol): generation of $Co(OH)_2$ was 0.677 mol, resulting in 62.9 g.
(4) $Mn(OH)_2$ (M = 88.952 g/mol): generation of $Co(OH)_2$ was 1.0 mol, resulting in 88.9 g.
(5) $(Ni/Co/Mn(OH)_2$: generation of $(Ni/Co/Mn(OH)_2$ was 159.1 + 62.9 + 88.9 = 311 g.
(6) Na_2SO_4 (M = 142.04 g/mol): generation of Na_2SO_4 was 1.716 + 0.677 + 1.0 = 3.392 mol, resulting in 481.9 g.

Route III

A. Energy consumption

1. Bond activation process

Electricity I: The total power of the ball-milling furnace used in this study was 0.3 kW (1200 rpm). The energy consumption for 12 h operation of the device at 800 rpm was about 800/1200 × 0.3 × 12 = 2.4 kW·h.

2. Pyrometallurgical process

Electricity II: The total power of the atmosphere furnace used in this study was 10.0 kW (1200 °C), the amount of solid sample that can be sintered in the atmosphere furnace was about 2.5 kg each time. First, the temperature was increased from room temperature to 550 °C at a heating rate of 10°C/min (about 52 min, 0.87 h), then holding the reaction temperature at 550 °C for 10 min (0.17 h). The energy consumption was 10/5 × 0.87 + 10 × 800/1200 × 0.17 = 1.85 kW·h.

3. Dissolution separation

Electricity III: MVR evaporation method for 1 ton of water requires the power about 23-70 kW·h, and it is assumed that it is 50 kW·h here. The solubility of Li_2CO_3 at room temperature is 1.33 g/100 g of water, so it takes 9.51 kg of water to obtain 126.5 g of Li_2CO_3. The power consumption of 9.51 kg of water with MVR evaporation technology is 50 × 9.51/1000 = 0.48 kW·h.

4. Acid Leaching process

Electricity IV: The power of the magnetic stirring device adopted was 0.25 kW (900 rpm). When the stirring speed was 200 rpm, the operating power of the magnetic stirring device was 0.25 × 200 ÷ 900 = 0.056 kW. The run time of the leaching reaction was set to 0.5 h. The total electricity consumption was 0.056 × 0.5 = 0.028 kW·h.

B. Chemicals consumption and obtained products

For 1.0 kg spent NCM523 battery, 392.84 g of cathode materials (3.01% C (11.7g, M = 12 g/mol), 6.03% Li (23.69 g, M = 6.94 g/mol, n = 3.413 mol), 25.64% Ni (100.72 g, M = 58.69 g/mol, n = 1.716 mol), 10.15% Co (39.87 g, M = 58.69 g/mol, n = 0.677 mol), and 13.98% Mn (54.92 g, M = 54.938 g/mol, n = 1.0 mol)) were obtained.

1. Bond activation process

(1) C (M = 12.01 g/mol): addition/consumption of C was 5% mass ratio of NCM523+C mixture, that was 20.7 g.

2. Pyrometallurgical process

All the data in the pyrometallurgical process were derived from the chemical reaction described as Eq.S4.

(1) CO_2 (M = 44 g/mol): emission of CO_2 was 20.7 ÷ 12.01 = 1.72 mol, resulting in 75.8 g.

(2) Li_2CO_3 (M = 73.89 g/mol): generation of Li_2CO_3 was 3.413 mol ÷ 2 × 1 = 1.71 mol, resulting in 126.5 g.

(3) NiO (M = 74.693 g/mol): generation of NiO was 1.716 mol, resulting in 128.2 g.
(4) CoO (M = 74.93 g/mol): generation of CoO was 0.677 mol, resulting in 50.7 g.
(5) MnO (M = 70.937 g/mol): generation of MnO was 1.0 mol, resulting in 70.9 g.

3. Acid Leaching process

All the data in the acid leaching process were derived from the chemical reaction described as Eq.S2.

(1) H_2SO_4 (M = 98.08 g/mol): consumption of H_2SO_4 was 1.716 + 0.677 + 1.0 = 3.392 mol, resulting in 332.8 g.
(2) $NiSO_4$ (M = 154.76 g/mol): generation of $NiSO_4$ was 1.716 mol, resulting in 265.6 g.
(3) $CoSO_4$ (M = 154.996 g/mol): generation of $CoSO_4$ was 0.677 mol, resulting in 104.9 g.
(4) $MnSO_4$ (M = 151 g/mol): generation of $MnSO_4$ was 1.0 mol, resulting in 151 g.

4. Chemical precipitation

All the data in the chemical precipitation were derived from the chemical reaction described as Eq.S3.

(1) NaOH (M = 40 g/mol): consumption of NaOH was (1.716 + 0.677 + 1.0) × 2 = 6.784 mol, resulting in 271.4 g.
(2) $Ni(OH)_2$ (M = 92.708 g/mol): generation of $Ni(OH)_2$ was 1.716 mol, resulting in 159.1 g.
(3) $Co(OH)_2$ (M = 92.95 g/mol): generation of $Co(OH)_2$ was 0.677 mol, resulting in 62.9 g.
(4) $Mn(OH)_2$ (M = 88.952 g/mol): generation of $Co(OH)_2$ was 1.0 mol, resulting in 88.9 g.
(5) $(Ni/Co/Mn(OH)_2$: generation of $(Ni/Co/Mn(OH)_2$ was 159.1 + 62.9 + 88.9 = 311 g.
(6) Na_2SO_4 (M = 142.04 g/mol): generation of Na_2SO_4 was 1.716 + 0.677 + 1.0 = 3.392 mol, resulting in 481.9 g.
